# Transcriptome and Flavonoids Metabolomic Analysis Identifies Regulatory Networks and Hub Genes in Black and White Fruits of *Lycium ruthenicum* Murray

**DOI:** 10.3389/fpls.2020.01256

**Published:** 2020-08-14

**Authors:** Tingting Li, Yunfang Fan, Huan Qin, Guoli Dai, Guoxiu Li, Yanlong Li, Jingjin Wang, Yue Yin, Fang Chen, Xiaoya Qin, Youlong Cao, Lin Tang

**Affiliations:** ^1^ Key Laboratory of Bio-Resources and Eco-Environment of Ministry of Education, College of Life Sciences, Sichuan University, Chengdu, China; ^2^ Institute of Wolfberry Engineering Technology, Ningxia Academy of Agriculture and Forestry Sciences, Yinchuan, China; ^3^ National Wolfberry Engineering Technology Research Center, Yinchuan, China

**Keywords:** anthocyanin, fruits, flavonoid, *Lycium ruthenicum*, metabolic profiling, transcriptome

## Abstract

*Lycium ruthenicum* Murry. is a highly nutritional cash crop due to its fruit abundant anthocyanins. To understand the complex metabolic networks underlying the color formation in black and white fruits of *L. ruthenicum*, we conducted transcriptome and flavonoid metabolic profiling to identify the candidate genes possibly involved in flavonoid biosynthesis. As a result, 147 flavonoids were identified and there was almost no anthocyanin in white fruits, while luteolin, kaempferol, and quercetin derivatives showed markedly higher abundance. Furthermore, applying weighted gene co-expression network analyses, 3 MYB, 2 bHLH, 1WRKY and 1 NAC transcription factor, associated with anthocyanin biosynthesis were identified. A bHLH transcription factor, *LrAN1b* showed the greatest correlations with anthocyanin accumulation with no expression in white fruits. In addition, gene function analysis and qRT-PCR experiments identified a new activated anthocyanin MYB transcription factor designed as *LrAN2-like*. Yeast two-hybrid and transient tobacco overexpression experiments showed that *LrAN1b* could interact with *LrAN2-like* and *LrAN11* to form MBW complex to activate the anthocyanin pathway. The yeast one-hybrid experiment indicated that *LrAN2-like* bonded anthocyanin structural gene *LrDFR* and *LrANS* promoters. Heterologous expression of *LrAN1b* in tobacco can significantly increase the anthocyanin content of tobacco florals and capsules, and activate anthocyanin synthesis related genes. Taken together, an anthocyanin regulatory network model in *L. ruthenicum* fruit was proposed firstly and we speculate that the white fruit phenotype was due to abnormal expression of *LrAN1b*. The findings provide new insight into the underlying mechanism of flavonoids, laying the foundation for future functional and molecular biological research in *L. ruthenicum*.

## Introduction

Anthocyanins are an important subclass of water-soluble flavonoids pigments that can give plants a bright red to blue color. Anthocyanin has many beneficial functions for human health, such as antioxidant activity (Jain et al., 2008) inhibition of tumor cells and prevention of chronic human diseases (Butelli et al., 2008). Anthocyanin is a downstream product of the flavonoid pathway. It takes 4-coumaroyl-CoA and malonyl-CoA as substrates; is synthesized by a series of enzymes encoded by the structural genes chalcone synthase (CHS), chalcone isomerase (CHI), flavonoid 3-hydroxylase (F3H), flavonoid 3′-hydroxylase (F3’H), flavonoid 3′5′-hydroxylase (F3’5’H), dihydroflavonol 4-reductase (DFR) and anthocyanidin synthase (ANS); and is transported to vacuoles after glycosylation, methylation, and acylation (Tanaka et al., 2010). In recent years, these structural genes and modification- and transport-related genes are mainly regulated by R2RMYB transcription factors, basic helix-loop-helix proteins (bHLH), and WD40 protein (MBW complexes) (Koes et al., 2005). Among them, MYB and bHLH can specifically bind cis-elements of structural gene promoter regions named MRE (MYB-recognizing element) and BRE (bHLH-recognizing element) (Zhu et al., 2015). In contrast, some studies have demonstrated that *Arabidopsis* *CPC* and *MYBL2* can inhibit anthocyanin biosynthesis by inhibiting the assembly of ternary MBW (Matsui et al., 2008; HF et al., 2009). In addition, other transcription factors also participate in anthocyanin regulation including WRKY protein, NAC protein, etc (Morishita et al., 2009; Verweij et al., 2016). The molecular mechanism of fruit color mutation has always been an important issue for researchers, because fruit color is closely related to the nutritional value, appearance, and taste of the fruit (Paauw et al., 2019). Anthocyanin pathway biosynthesis or transcription regulation encoding gene mutations are related to color phenotype. In red pulp apples, multiple repeats of the transcription factor MYB10 promoter fragment result in automatic regulation of transcription factors (Espley et al., 2009). In strawberries, sequence changes in the upstream regulatory region of FnMYB10 resulted in low expression levels of the FnMYB10 gene, which most likely resulted in the white fruit phenotype of *F. nilgerrensis* (Zhang J. et al., 2020). For *L. ruthenicum*, an important economic crop as a source of anthocyanins, genetic control of the fruit color and exploration of germplasm resources are of particular concern.

*Lycium ruthenicum* Murry. is a small bush belonging to the Solanaceae family the fruits of which are used widely as ethnic medicine and nutraceutical food (Hu et al., 2014). The genus *Lycium* (Solanaceae) approximately 80 species and widely grows in arid to semi-arid environments of the temperate zones, and it is distributed worldwide in very different habitats (Levin and Miller, 2005). The fruits behave as functional components and are beneficial for human health; their compounds are mainly polyphenols such as flavonols, anthocyanins, and catechins, strong natural antioxidants (Wang et al., 2018). Modern medical experiments show that *L. ruthenicum* fruit extracts can resist pancreatic ductal adenocarcinoma cell activity (Zhang S. et al., 2019) and cure diabetic cardiomyopathy (Xue et al., 2014). Recent evidence suggests that the main active ingredient in this species is petunidin-3-*O*-rutinoside (trans-p-coumaroyl)-5-*O*-glucoside (Zheng et al., 2011). In the latest study, 49 compounds including anthocyanins, alkaloids, hydroxycinnamic acid derivatives, flavonoids and amino acids were initially identified in the four developmental stages of *L. ruthenicum* fruit (Yang et al., 2019). In recent years, a wild white berry variety without anthocyanin of *L. ruthenicum* has been found in Qinghai Province China (Yin-Yan et al., 2017). However, there are fewer report illustrating the ;secondary metabolites and molecular mechanism in the white fruits of *L. ruthenicum*. Previous research has identified the anthocyanin-related structural genes and transcription factors including MYB protein (*LrAN2* and *LrMYB113*), bHLH protein (*LrJAF13* and *LrAN1b*), and WD40 protein (*LrAN11*) in *L. ruthenicum* (Zeng et al., 2014). Recent studies claim that the functional diversity and high expression levels of *LrAN2* may be responsible for the high content of anthocyanins in *L. ruthenicum* fruits (Zong et al., 2019).

In this study, to obtain insights into the changes of flavonoid metabolites and transcriptional regulation of the anthocyanin synthetic pathways in black and white fruits of *L. ruthenicum*. We performed ﬂavonoid metabolites of mature fruits of two colors using liquid chromatography tandem mass spectrometry (LC-MS/MS). Transcriptome sequencing of two fruit colors was performed on five distinct fruit development stages, and differentially expressed flavonoid and anthocyanin structural genes were identified. Furthermore, we obtained anthocyanin-related co-expression gene modules and screened some key synthetic and regulatory genes involved in anthocyanin synthesis by WGCNA. Accordingly, the expression levels of biosynthetic genes and regulatory genes of two different fruit colors were analyzed by quantitative real-time polymerase chain reaction (qRT-PCR). Here, first, we used yeast two-hybrid and tobacco transient expression experiments to prove that the candidate genes *LrAN2-like* and *LrAN1b* can interact and combine with AN11 to form an MBW complex. In addition, the *LrDFR* and *LrANS* gene promoters of kiwifruit were used in the plant *in vivo* screening test to identify R2R3 MYB (*LrAN2-like*) that binds Lr. We characterized the *LrAN1b* gene in Lr and analyzed its overexpression in model plant tobacco. The results show that *LrAN2-like* and *LrAN1b* can interact and act on anthocyanin accumulation. This work not only interprets the metabolomic flux change of flavonoids in *L. ruthenicum* fruits with black or white colors but also further refines the *L. ruthenicum* anthocyanin regulatory network.

## Materials and Methods

### Plant Material and Sampling

**Figure 1:**
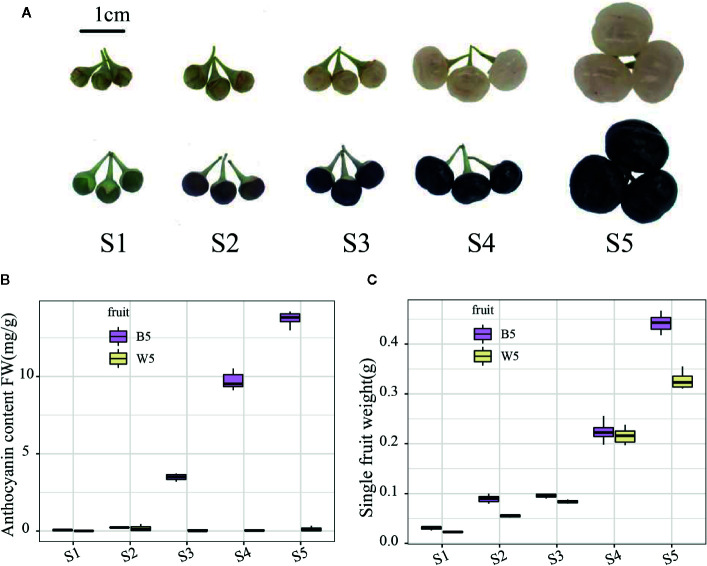
Fruit colors and phenotypic characterization of different developmental stages between two varieties. **(A)** Fruit colors of the two varieties at S1 (3 days before breaker); S2 (breaker); S3 (3 days after breaker); and S4 (6 days after breaker); and S5 (10 days after breaker). **(B)** Anthocyanin content in S1–S5. **(C)** The average fruit weight in S1–S5. Three independent biological repeats were used for the determination of anthocyanin. Fruit weight is the average weight of every ten fruits, with three biological repeats.

Two *L. ruthenicum* varieties were collected at five different developmental stages (S1, S2, S3, S4, and S5) from the *Lycium* resource nursery of the Ningxia Academy of Agricultural and Forestry Sciences in Xixia district, the Ningxia Hui Autonomous Region, China. S1–S5 are shown in **Figure 1A**, and the method of time selection referred to the articles of Zeng et al. (2014) S1 refers to the small and green fruits 3 days before breaker; S2 refers to the color breaker stage of fruits; S3 refers to period of complete discoloration (3 days after breaker), S4 refers to the early expansion and deepening of pigmentation (6 days after breaker), and S5 refers to the period of complete maturity (10 days after breaker). We collected fruits from three *Lycium ruthenicum* Murray trees separately, and each tree collected about 30 fruits from July to August, 2018. The fruits on each tree are treated as an independent biological repeat, so we have three independent biological repeats for each kind fruit. Similarly, for the metabolome samples, we collected about 50 mature fruits (S5 period) from the three trees for analysis respectively. The fruit of each tree was treated as an independent biological repeat, for a total of three biological repeats. Thus, all data were obtained based on three independent biological replicates. All fruits were snap-frozen in liquid nitrogen, and then kept at -80°C for subsequent metabolite extraction, transcriptome sequencing, and real-time PCR analysis.

### Metabolite Extraction

Samples flavonoid content were performed according to previously methods (Chen et al., 2013). The freeze-dried fruit was crushed using a mixer mill (MM 400, Retsch) with a zirconia bead for 1.5 min at 30 Hz. 100 mg powder was weighted and extracted overnight at 4°C with 1.0 ml 70% aqueous methanol. Following centrifugation at 10,000 g for 10 min, the extracts were absorbed (CNWBOND Carbon-GCB SPE Cartridge, 250mg, 3 ml; ANPEL, Shanghai, China, www.anpel.com.cn/cnw) and filtrated (SCAA-104, 0.22μm pore size; ANPEL, Shanghai, China, http://www.anpel.com.cn/) before LC-MS analysis. Three biological replicates and three technical assays for each biological repetition were analyzed.

### HPLC Conditions and ESI-Q TRAP-MS/MS

The sample extracts were analyzed using an LC-ESI-MS/MS system (HPLC, Shim-pack UFLC SHIMADZU CBM30A system, www.shimadzu.com.cn/; MS, Applied Biosystems 6500 Q TRAP, www.appliedbiosystems.com.cn/). The analytical conditions of HPLC and MS were referred to previous method (Chen et al., 2013). HPLC: column, Waters ACQUITY UPLC HSS T3 C18 (1.8 µm, 2.1 mm*100 mm); solvent system, water (0.04% acetic acid): acetonitrile (0.04% acetic acid); gradient program,100:0V/V at 0min, 5:95V/V at 11.0min, 5:95V/V at 12.0min, 95:5V/V at 12.1min, 95:5V/V at 15.0 min; flow rate, 0.40 ml/min; temperature, 40°C; injection volume: 2 μl. The effluent was alternatively connected to an ESI-triple quadrupole-linear ion trap (Q TRAP)-MS.

Linear ion trap (LIT) and triple quadrupole (QQQ) scans were acquired on a triple quadrupole-linear ion trap mass spectrometer (Q TRAP), API 6500 QTRAP LC/MS/MS System, equipped with an ESI Turbo Ion-Spray interface, operating in a positive ion mode and controlled by Analyst 1.6 software (AB Sciex). The ESI source operation parameters were as follows: ion source, turbo spray; source temperature 500°C; ion spray voltage (IS) 5500 V; ion source gas I (GSI), gas II(GSII), curtain gas (CUR) were set at 55, 60, and 25.0 psi, respectively; the collision gas(CAD) was high. Instrument tuning and mass calibration were performed with 10 and 100 μmol/L polypropylene glycol solutions in QQQ and LIT modes, respectively. QQQ scans were acquired as MRM experiments with collision gas (nitrogen) set to 5 psi. Declustering potential (DP) and collision energy (CE) for individual MRM transitions was done with further DP and CE optimization. A specific set of MRM transitions were monitored for each period according to the metabolites eluted within this period.

### Flavonoids Identification and Quantification

Qualitative analysis of primary spectrum and secondary spectrum data of mass spectrometry detection, based on self-built database MWDB (metware database) and public database of metabolite information. The isotope signal and the K+ ion, Na+ ion, and NH4+ ion repetitive signal removed when some substances were quantified. The metabolite structure analysis refers to MassBank (http://www.massbank.jp/), KNAPSAcK (http://kanaya.naist.jp/KNApSAcK/), HMDB (http://www.hmdb.ca/) (Wishart et al., 2013), MoTo DB (http://www.ab.wur.nl/moto/) and METLIN (http://metlin.scripps.edu/index.php) (Zhu et al., 2013) mass spectrometry Public database.

Metabolite quantification is accomplished using multiple reaction monitoring mode (MRM) analysis using triple quadrupole mass spectrometry. The quadrupole first screens the precursor ions of the target substance and excludes ions corresponding to other molecular weight substances to preclude interference. The precursor ions are fragmented after induced ionization in the collision chamber to form a lot of fragment ions. The fragment ions are then filtered through a triple quadrupole to select a desired fragment ion to eliminate interference from non-target ions. After obtaining the metabolite mass spectrometry data of different samples, integrate the peak area of the mass spectrum peaks of all substances, and integrate and correct the peaks of the same metabolite in different samples (Fraga et al., 2010).

### RNA-Seq Analysis

The total RNA was extracted from frozen fruits, the mRNA library of each sample was constructed and sequenced in the Illumina HiSeq platform, and paired-end reads were generated. There are three biological repeats in each sample. Raw data of fastq format were first processed through in-house Perl scripts. Clean data were obtained by removing reads containing adapter, reads containing ploy-N and low-quality reads from raw data. Transcriptome assembly was accomplished based on left.fq and right.fq using Trinity (r20140413p1) (MG et al., 2011). Gene function was annotated based on the following databases: Nr (NCBI non-redundant protein sequences) (v0.2.28) (Nguyen et al., 2007); Nt (NCBI non-redundant nucleotide sequences) (v2.2.28+) (Nguyen et al., 2007); Pfam (Protein family) (HMMER 3) (Finn et al., 2008); KOG/COG (Clusters of Orthologous Groups of proteins) (v0.2.28); Swiss-Prot (A manually annotated and reviewed protein sequence database) (v0.2.28); KO (KEGG Ortholog database) (r140224) (Kanehisa et al., 2008); and GO (Gene Ontology) (b2g4pipe_v2.5) (Götz et al., 2008). The gene expression levels were estimated by RSEM (v1.2.15) (Bo and Dewey, 2011) for each sample. A differential expression analysis of two samples was performed using the DEGseq R package (1.12.0) (Likun et al., 2010). The p-value was adjusted using the q value (Storey and Tibshirani, 2003), qvalue<0.005 & |log2 (fold change) |>1 was set as the threshold for significantly differential expression. The Gene Ontology (GO) enrichment analysis of the differentially expressed genes (DEGs) was implemented by the GOseq R package (1.10.0) (MD et al., 2010) based Wallenius non-central hypergeometric distribution. We used KOBAS software (v2.0.12) (Mao et al., 2005) to test the statistical enrichment of differential expression genes in KEGG pathways. The sequences of the DEGs were blasted to the genome of a related species (the protein interaction of which exists in the STRING database: http://string-db.org/) to obtain the predicted PPI of these DEGs. Then, the PPI of these DEGs was visualized in Cytoscape (v3.7.2) (Shannon et al., 2003). The raw sequence data reported in this paper have been deposited in the Genome Sequence Archive (Wang et al., 2017) in National Genomics Data Center (Zhang Z. et al., 2020), Beijing Institute of Genomics (China National Center for Bioinformation), Chinese Academy of Sciences. The accession number(s) is CRA002484 that are publicly accessible at https://bigd.big.ac.cn/gsa/s/VCuA768g.

### Co-expression Network Construction of Metabolome and Transcriptome

A weighted gene co-expression network analysis (WGCNA) was analyzed using the WGCNA R package (v1.68) (Langfelder and Horvath, 2008) based on FPKM values. Network construction and module identification were conducted using the topological overlap measure (TOM). The calculation parameters “soft thresholding power” =14, “minModuleSize” = 30 and “mergeCutHeight” = 0.25 were selected for analysis of the transcriptome data sets. The modules were used to calculate the relationships among modules, anthocyanin content, and representative genes in the 10 samples. The crucial module networks were visualized using Cytoscape with threshold = 0.25.

### Sequence Alignment and Phylogenetic Analysis

To investigate the mechanism regulating anthocyanin biosynthesis in *L. ruthenicum*, the deduced amino acid sequences of MYBs: LrAN2, LrAN2-like, MYB113, bHLHs: LrAN1b, and LrJAF13 were obtained from transcriptome data. 40 MYBs and 31 bHLHs of other species were downloaded from NCBI database. The amino acids of the MYB proteins and the conserved domains of bHLH proteins were engaged to perform phylogenetic analysis using MEGA (version 6) with the neighbor-joining statistical method and 1000 bootstrap replicates.

### Yeast Two-Hybrid Analysis

According to the Matchmaker^®^ Gold Yeast Two-Hybrid System (Clontech, HTTP://www.clontech.com/), the Full-length cDNAs of the candidate genes *LrAN2-like*, *LrAN1b*, *LrJAF13*, *LrAN11* were respectively cloned in the pGBKT7 and pGADT7, and then co-transformed into the yeast strain AH109. The primers listed in **Supplemental Table 1**. The transformants were selected on SD/-Leu/-Trp medium and tested on SD/-Leu/-Trp/- His/-Ade medium by increasing amounts of 3-amino-1,2,4-triazole (3-AT) and X-α-Gal. Meanwhile, pGADT7 and pGBKT7 Co-transformed with candidate genes as negative control. Growth was scored after 2 d at 30°C.

### Yeast One-Hybrid Analysis

The method of Y1H based on previous report (Shim et al., 2013). The *LrAN2-like* CDS recombinded with GAL4 activation domain of pGADT7. A DNA fragment consisting of three copies of the DFR (-804 to -835) and ANS (-1385 to -1415) promoter sequence containing the R2R3-MYB core binding domain sequence was chemically synthesized and homologous recombination into the pHis2 vector. The AD- *LrAN2-like*, were respectively Co-transformated with pHis2-DFR^MRE^, pHis2-ANS^MRE^and grown on the SD/- Leu/-Trp medium. Then spot the yeast suspension on the SD/-Leu/-Trp/-His medium, with or without 3-AT (0 or 50 mM). The pGADT7 and four pHis2-DNA fragments respectively Co-transformated for the negative control. The primers listed in **Supplemental Table 1**.

### Transient Over-Expression in Tobacco Leaves

The vector pEAQ-HT with 35S: *LrAN2-like* and 35S: *LrAN1b* construct were transformed into Agrobacterium tumefaciens strain GV3101 by heat shock method followed by incubation on plate before infiltration. The cultures of Agrobacterium were resuspended to OD600 reached about 0.75 using buffer (10 mM MES, 10 mM MgCl2, 200 mM acetosyringone, pH 5.6) before infiltration to the leaves. *N. tabacum* plants were grown in glasshouse with 26°C, 16 h light, and 8 h dark conditions. The leaves of 6-week-old N. tabacum with three biological replicates were used for infiltration. The Agrobacterium cultures with empty vector pEAQ-HT acted as a negative control. All primers used for this experiment were listed in **Table S1**. Leaves were harvested 7 days after infiltration to further phenotypic observation, anthocyanin determination and RT-qPCR. All samples were analyzed from at least three biological replicates.

### Plant Expression Vectors and Stable Tobacco Transformation

In order to verify the function of *LrAN1b*, the ORF sequence for *LrAN1b* was inserted into pCM1307. The primers were listed in **Table S1**. Thus, *LrAN1b* was promoted by the 35S promoter. The recombinant or empty vector plasmid was introduced into GV3101 by the freeze–thaw method.

Tobacco plants (*N. tabacum*) were transformed with Agrobacterium as described previously (Horsch et al., 1985). The transformed tobacco plants were screened using 25 mg/L^−1^ hygromycin antibiotic, which was used as the plant selective marker. Two transgenic tobacco lines were obtained and the transformation was confirmed using qRT-PCR, which designed OE#1 and OE#2. The seeds of the transgenic plants were harvested separately. The next stage of analysis used T1 generation transgenic plants of overexpressing OE- *LrAN1b* that showed obvious color changes in the florals and capsules.

### Measurement of Total Anthocyanin Content

Total anthocyanin content was determined using revised method referring to the previous method (Shin et al., 2007). 0.1g sample was ground into powder by liquid nitrogen and incubated in 600µL of extraction buffer (methanol containing 1% HCl) overnight at 4°C in the dark. After extraction, 400 µL of water and 400 µl of chloroform were added to each sample to remove chlorophyll. Then samples were centrifugated at 14,000 g for 5min at 4°Cto sediment the plant material and absorbances were read at 530 and 657 nm. The quantity of anthocyanin was determined A530 – 0.33×A657, and each sample was extracted and measured in three independent experiments.

### QRT-PCR Analysis

High-quality total RNA was used for reverse transcription PCR using the Takara reverse transcription kit (PrimeScript™ RT reagent kit, Dalian, China). The qRT-PCR 20 µL reaction system was constructed using the AceQ qPCR SYBR Green Master Mix (Vazyme, Nanjing, China). All primers used in this study are listed in **Supplementary Table S1**. Real-time assays were performed with SYBR Green Dye using CFX96 Touch™ Real-Time PCR (Bio-Rad, Hercules, CA, USA) detecting platform. The primer amplification effectiveness was analyzed using CFX Manager™ (v3.0) software and the expression of genes was calculated by the 2^- ΔΔCt^ method. The values were expressed as means ± standard error (SE). Three biological replicates and technical duplication were used in RT-qPCR analysis.

### Statistical Analysis and Data Availability

All experimental data from three independent biological replicates were subjected to a one-way analysis of variance (ANOVA). Significant differences were calculated by the Student’s t-test: tests (p<0.05) using SPSS 22.0 Statistics (SPSS Inc., Chicago, IL, USA).

## Results

### Phenotypic Characterization of Two-Color Fruits of *L. ruthenicum* in Different Developmental Stages

*L. ruthenicum* fruits of two different colors were investigated to study the phenotypic characterization. For black-fruited *L. ruthenicum*, fruit development was separated into green (S1), turning (S2), purple (S3), black (S4), and fully inflated black (S5) stages (**Figure 1A**). In contrast, the white-fruited *L. ruthenicum* turned white at the S2 stage and changed from green to transparent-white fruit without pigment accumulation (**Figure 1A**). The further fruit weight results indicated no difference in the size of the two fruits from S1 to S4; however, in the S5 period the black fruit was larger than the white berry by a difference of approximately 0.1425 g (**Figure 1C**). Moreover, the anthocyanin content analysis revealed that the anthocyanin content of black fruits increased sharply in the S3 period, reaching the highest in S5 (full maturity) (13.47 mg g^−1^ of FW). In contrast, almost no anthocyanins were detected in the white fruits, which is compatible with the phenotype (**Figure 1B**). The results show that the black fruit of *L. ruthenicum* may be a good choice as an anthocyanin supplement for consumers, highlighting the prodigious differences in anthocyanin accumulation in black and white fruits of *L. ruthenicum*.

### Identification of Flavonoids in the Fruits of Two *L. ruthenicum* Varieties

**Figure 2:**
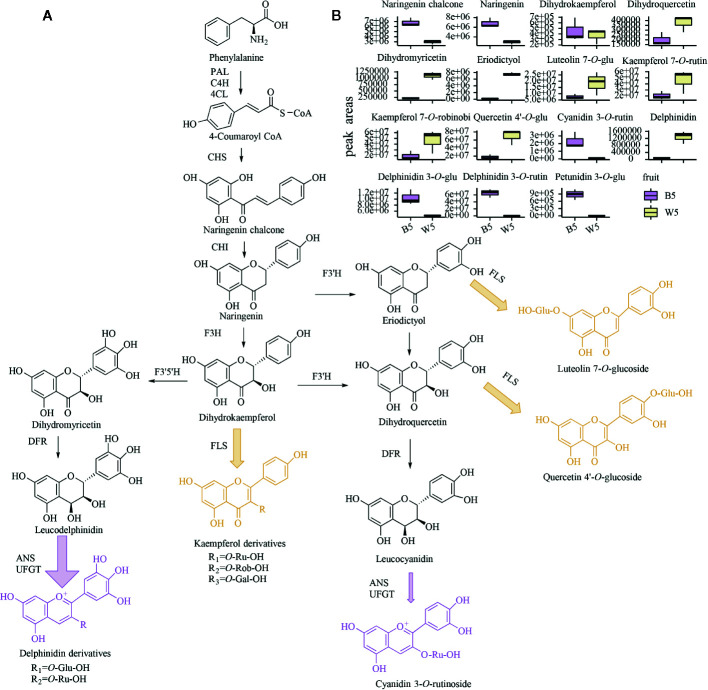
Flavonoid metabolome of two fruits at S5. **(A)** a diagram of the putative anthocyanin metabolic process in black or white fruits; purple represents the main flavonoid component of black fruit, and yellow represents the main flavonoid substance that metabolizes white berries. **(B)** Box plots of the relative content of 14 major flavonoids in two fruits; center lines denote medians, bounds of boxes denote first and third quartiles. HPLC analyses were performed on three biological replicates.

To compare the differences in flavonoid metabolites in two *L. ruthenicum* varieties, B5 (S5 of black fruit) and W5 (S5 of white fruit) were subjected to LC-ESI-Q- TRAP-MS/MS analysis due to the most abundant metabolite content. The fruits of W5 are purple-black and rich in pigments, while the fruits of B5 are white (**Figure 1A**). In this work, 147 flavonoid metabolites were identified and quantified in two *L. ruthenicum* fruits (**Tables S2** and **S3**). The principal component analysis (PCA) analysis showed that B5 and W5 were separated in the PC1×PC2 score plots with 73.12% of PC1 and 14.69% of PC2 (**Figure S1**). The detected flavonoid species mainly included 8 catechin derivatives, 15 anthocyanins, 41 flavones, 30 flavone *C*-glycosides, 30 flavonols, 19 flavanones, and 6 isoflavones. Furthermore, we set |Log2 (fold change) |≥2, p-value < 0.05, and VIP (variable importance in the project) ≥ 1 as the thresholds for differentially accumulated flavonoids. All differential metabolites are integrated in **Figure S2** and **Table S4**. Furthermore, the box diagrams of the main accumulated components in *L. ruthenicum* are depicted in **Figure 2B**. B5 was twice as high as W5 in the main substances upstream of flavonoid metabolism, naringenin chalcone and naringenin. As is known, naringenin, a key substance in flavonoid biosynthesis, is catalyzed to form downstream of DHK (Dihydrokaempferol), DHM (dihydromyricetin), DHQ (Dihydroquercetin) by a series of enzymes (Tanaka et al., 2010). The results indicate that B5 has more upstream substrates to produce downstream flavonoids compared with W5. Otherwise, DHK had little difference in the component accumulation of the two fruits, and DHM and DHQ of W5 were 3 times higher than B5 (**Figure 2B**). These results suggest that although B5 has more upstream substrate naringenin chalcone and naringenin to produce downstream flavonoids, B5 also has more DHM and DHQ consumed to synthesize downstream anthocyanins compared with W5.

Previous research shows that 37 anthocyanins have been detected from *L. ruthenicum* fruits, including derivatives of peonidin, petunidin, pelargonidin, cyanidin, malvidin, and delphinidin (Wang et al., 2018). In this study, 15 kinds of anthocyanins were identified, including pelargonidin, cyanidin, delphinidin, peonidin, petunidin, malvidin, and rosinidin derivatives. Among these anthocyanins, delphinidin 3-*O*-rutinoside and petunidin 3-*O*-glucoside were the most significant and abundant metabolites; they accumulated in B5 but not in W5. The secondary mass spectrum of delphinidin 3-*O*-rutinoside is shown in **Figure S3A**. This finding is consistent with previous data finding that petunidin derivatives account for 95% of the total anthocyanins in the fresh fruit (Zheng et al., 2011). Moreover, a small amount of rosinidin *O*-hexoside, malvidin 3-*O*-glucoside, malvidin 3-*O*-galactoside, and peonidin *O*-malonylhexoside was only detected in W5. This finding suggests that very trace amounts of anthocyanins are still synthesized in W5. Moreover, although delphinidin was 4 times higher than in B5, W5 did not produce more delphinidin derivatives (**Figure 2B**). It is likely that the delphinidin of W5 lacked modified enzymes to synthesize downstream metabolites.

Similar to anthocyanins, other flavonoids also have extraordinary antioxidant, antitumor, antibacterial, and anticancer activities (Falcone Ferreyra et al., 2012). In our study, 23 flavonoid metabolites were found only in black fruits and not detected in white fruits. These flavonoid metabolites include 3 ‘, 4’, 5’-dihydrotricetin *O*-hexosyl-*O*-hexoside, chrysoeriol *O*-hexosyl-*O*-rutinoside, tricin *O*-rhamnoside, acacetin *O*-acetyl hexoside, chrysin, and pinocembrin in lignin metabolism, 5 myricetin derivatives, 3 catechin derivatives, 3 luteolin glycoside, 4 apigenin glycosides, 2 hesperidin glycosides, and two isoflavones of hydroxygenistein and sissotrin. On the other hand, sakuranetin, kumatakenin, 6-*C*-hexosyl-apigenin *O*-hexosyl-*O*-hexoside, isosakuranetin, daidzin, and glycitin only accumulated in W5 and were not detected in B5. The results show that B5 and W5 only accumulated small amounts of isoflavones and proanthocyanidins, and there was not much difference between the two fruits. Furthermore, the decrease in flavonoids species in W5 may be to reduce the number of branches and synthesize more other flavonoids. Although there are fewer flavonol species in W5, the resulting peak area integration showed that the four most common and representative flavonols in *L. ruthenicum* fruits were kaempferol 3-*O*-rutinoside, kaempferol 3-*O*-robinobioside, quercetin 4’-*O*-glucoside and luteolin 7-*O*-glucoside. The secondary mass spectra of the main four flavonoids in white fruits are shown in **Figures S3B, C,** and **D**, respectively. These four flavonols were detected at a 2-3-fold higher level in W5 than in B5 (**Figure 2B**). In addition, to determine why certain steps of the anthocyanin biosynthetic pathway in W5 were blocked, the intermediates involved in the metabolic process and their main branches were compared. A schematic diagram of the anthocyanin metabolism process and its core metabolites and enzymes in black and white *L. ruthenicum* fruits is shown in **Figure 2A**. The result indicated that purple compounds and arrows represented an accumulation of delphinidin derivatives and small amounts of cyanidin derivatives in B5. Yellow compounds and arrows indicate that several flavonols were accumulated in large amounts in W5. However, despite a sharp decrease in anthocyanins, W5 still contained all other core metabolites detected in B5 (**Figure 2A**). Notably, the concentrations of kaempferol, quercetin and luteolin derivatives detected in W5 were significantly higher than in B5 (**Figure 2B**). These results suggest that both W5 and B5 possess the same flavonoid upstream pathway, but the W5 anthocyanin pathway is blocked downstream of naringenin. Then, DHK, DHQ, DHM, and eriodictyol are catalyzed by FLS to synthesize more flavonols instead of continuing to synthesize anthocyanins. Despite no anthocyanin pigmentation in W5, flavonols preferably accumulate in W5. These findings are very similar to previous research on *grape hyacinth*; when anthocyanin synthesis is restricted in white flowers, upstream flux must flow into other branches of the flavonoid pathway (Lou et al., 2014).

### RNA-Seq and Assembly

To understand the molecular basis of fruit color polymorphism in *L. ruthenicum*, three biological replicates from each of five developmental stages of black fruits and white fruits of *L. ruthenicum* were used to construct cDNA libraries for high-throughput sequencing, including B1, B2, B3, B4, B5, W1, W2, W3, W4, and W5. After cleaning and quality assessment of the libraries, each library generated 47,356,860–73,227,696 clean reads of sequencing error rates <0.01% (**Table S5**). These reads were assembled into 274,634 unigenes with a mean size of 1,190 bp and an N50 of 1,860 bp (**Figure S4A**). The number of the unigene length distributions ranging from 500-2,000 was 140,901(**Figure S4B**), indicating that the proportion of full-length gene assembly in this study was sufficiently high for our further analysis. Because no genomic information is available for *L. ruthenicum*, to obtain comprehensive gene function information, all unigenes were blasted to seven databases including Nr, Nt, Pfam, KOG/COG, Swiss-prot, KEGG, and GO. Finally, 188,308 unigenes were annotated, accounting for 68.56% of all unigenes (**Table S6**). According to the results of the Nr library comparison annotation, the species distribution chart showed that the unigenes had the highest homology to genes of *Solanum tuberosum* (28.2%), *Nicotiana sylvestris* (19.5%), *Nicotiana tomentosiformis* (19.3%) and *Solanum Lycopersicum* (14.3%), implying that the function of the gene of *L. ruthenicum* may be very similar to that of the main model plants of Solanaceae (**Figure S5**). A total of 31,302 DEGs were identified in Transcriptome with padj < 0.05 and log2|fold change| > 1, which is shown in the Venn diagram (**Figure S6A**). Compared with white fruits, upregulated differential genes in black fruits are described by heat maps (**Figure S6B**). The DEGs were mainly divided into three clusters: W1-W5 were divided into cluster1, B1 and B2 were divided into cluster2, and B3-B5 were divided into cluster3. This result indicates that, similar to the anthocyanin phenotype of fruits, the upregulated differentially expressed genes in black fruits are mainly in the B3-B5 period, whereas the early B1-B2 period has less of a difference.

### DEGs Involved in Flavonoid Metabolism Between Two Fruit Varieties

**Figure 3:**
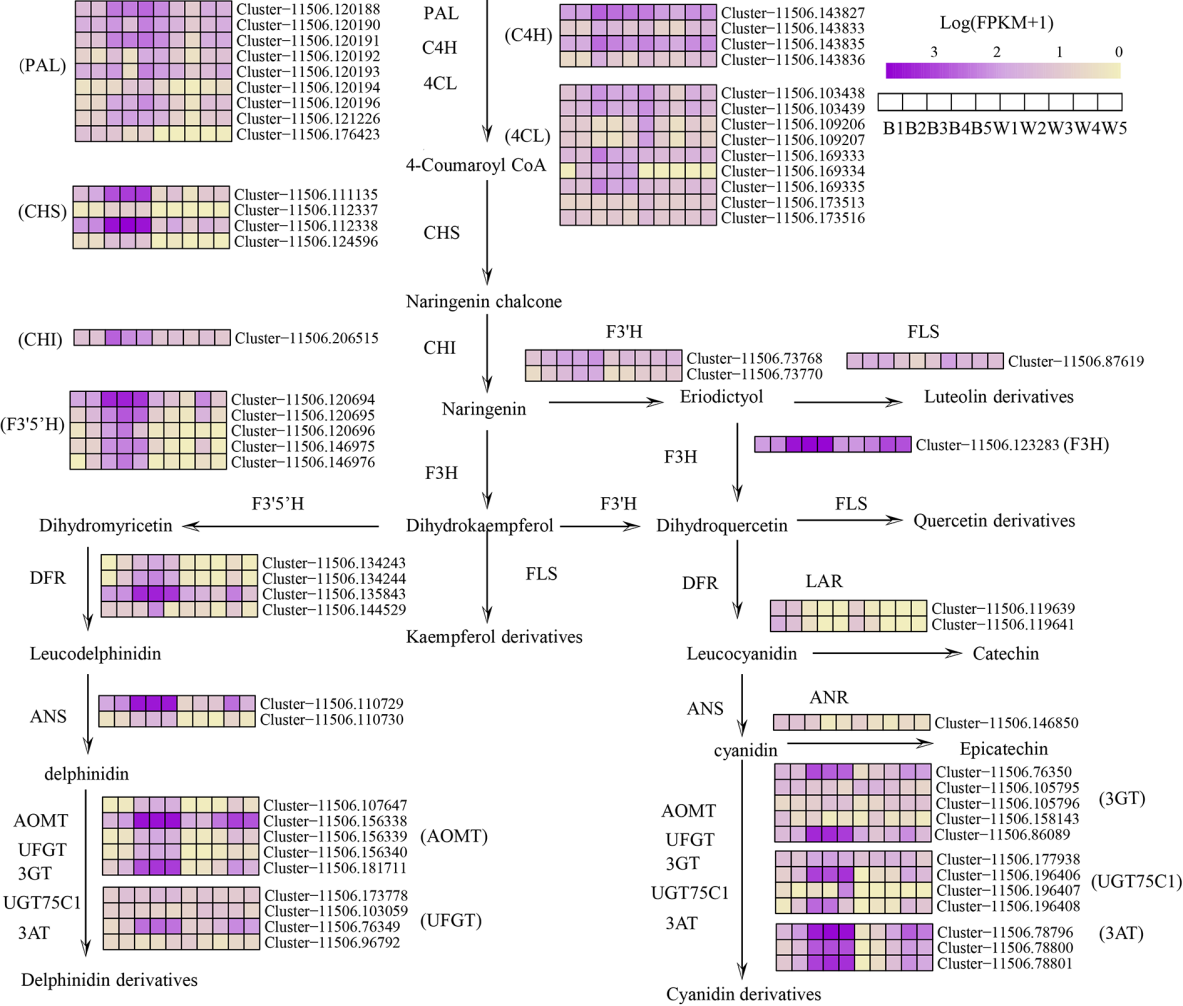
Simplified scheme and heat map of anthocyanin biosynthesis genes in fruit color development of L. ruthenicum. PAL, phenylalanine ammonia-lyase; C4H, cinnamate 4-hydroxylase;4CL,4-coumarate-CoAligase; CHS, chalcone synthase; CHI, chalcone isomerase; F3H flavonoid 3-hydroxylase; F3’H, flavonoid 3′-hydroxylase; F3’5’H, flavonoid 3′5′-hydroxylase; DFR, dihydroflavonol 4-reductase; ANS, anthocyanidin synthase; FLS, flavonol synthase; UFGT, UDP-glucose flavonoid 3-O-glucosyltransferase; 3GT, anthocyanidin 3-O-glucosyltransferase; UGT75C1, anthocyanidin 3-O-glucoside 5-O-glucosyltransferase; 3AT, anthocyanin acyltransferase; AOMT, Anthocyanin methyltransferase; LAR, leucoanthocyanidin reductase; ANR, anthocyanidin reductase. The color scale represents the log-transformed FPKM+1 value. Purple indicates high expression, and yellow indicates low expression.

To explore the molecular mechanisms leading to the differential fruit coloration in the two varieties, enzyme-encoding genes of the flavonoid pathways were investigated (**Figure 3** and **Table S7**). The analysis of transcriptome data revealed that 72 key candidates exerted direct influence over fourteen enzymes that were known to be involved in flavonoid and anthocyanin biosynthesis in our study (**Table S7**). The expression level of some phenylpropane biosynthetic genes, *LrPAL*, *LrC4L*, and *Lr4CH*, showed no differences in two fruit varieties (**Figure 3**). The result indicates no difference between the two fruits in the early phenylpropane pathway. Moreover, both the transcriptome analysis and qRT-PCR showed that most of the anthocyanin biosynthetic pathway genes, *LrCHS*, *LrCHI*, *LrF3H*, *LrF3’5’H*, *LrDFR*, and *LrANS*, were significantly downregulated in white fruits (**Figures 3** and **5A**). The three enzymes of *LrCHS*, *LrCHI*, and *LrF3H* are important and are considered to be the restriction enzymes to the synthesis of flavonoids (Zhang et al., 2014). These results explain that the difference between black and white fruits starts from the flavonoid pathway, which is also consistent with the differences between naringenin and naringenin chalcone. On the other hand, compared with black fruits, there was no significant difference in the gene expression of transcripts *LrF3’H* and *LrFLS* in white fruits. However, most plants hydroxylate the B-ring using either *LrF3’H* to synthesize red cyanidin or *LrF3’5H* to form a purple delphinium (Zhang et al., 2014). This shows that more delphinium is produced in the black fruit than cyanidin. Previous studies have shown that *LrDFR* and *LrFLS* share the same upstream substrates (e.g., DHM, DHQ, and DHK) and take a large proportion of total flavonoids in plants (Huang et al., 2019). In black fruits, the rate-limiting enzyme *LrDFR* specifically binds DHQ to synthesize delphinidin and its derivatives and has little binding to other substrates. This is accordant with previous studies, which means that DFR can selectively catalyze substrates, which correlates with the amino acid 134 being related to the 134th amino acid (Liu et al., 2019). However, in white fruits extremely low expression of *LrDFR* can no longer combine with the substrate DHQ to synthesize anthocyanins. Therefore, more substrates of DHK, DHQ, and eriodictyol were separately synthesized flavonols by *LrFLS* in the white fruits. Our study shows that the downregulation of *LrDFR* in white fruits results in *LrFLS* synthesizing more flavonols than anthocyanins, which is similar to the findings for *grape hyacinth* (Lou et al., 2014).

**Figure 4:**
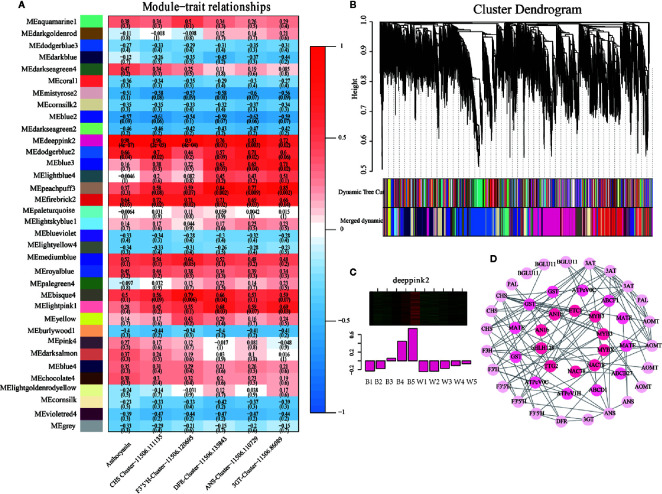
Weighted gene co-expression network analysis (WGCNA) of differentially expressed genes (DEGs) identified black and white fruits. **(A)** Module–trait correlations and the corresponding P-values are in parentheses. The left panel shows 35 modules. The color scale on the right indicates the correlation of module characteristics from -1 (blue) to 1 (red). The panel labeled “Anthocyanins” represents the biosynthetic properties of anthocyanins. The other panels represent changes in gene expression levels. **(B)** Hierarchical clustering tree showing 35 co-expressed gene modules. 23,642 DEGs are clustered into branches, and each module is represented by the main tree branch. The lower panel displays the module in the specified color. **(C)** Eigengene expression profile for the deeppink2 in different samples. The heat map above shows the expression profiles of all co-expressed genes in the module. The bar graph below shows the common expression pattern of genes co-expressed in the module. **(D)** Cytoscape representation of co-expressed anthocyanin metabolism-related genes with edge weights ≥0.25 in the “deeppink2” module. The correlation network diagram is divided into three layers, the outermost genes are phenylpropane, flavonoids, and anthocyanin synthesis genes, the second layer is anthocyanin transporter genes, and the most central layer is transcription factors.

The structure of anthocyanins is very unstable, and anthocyanins are generally glycosylated, methylated and acylated before transport to vacuole storage. In this study, the transcripts of anthocyanin glycosyltransferases (*LrUFGT*, *Lr3GT*, and *LrUGT75C1*), anthocyanin methyltransferase (*LrAOMT*) and anthocyanin acyltransferase (*Lr3AT*) were identified, and they were highly expressed in black fruits and only slightly expressed in white fruits (**Figures 3** and **4D**). As explained in a previous study, those glycosyl groups are usually linked to the carbon 3 position and sometimes the carbon 5 and carbon 7 position of the anthocyanin to increase the stability of the pigment (Zhang et al., 2014). *LrUFGT* and *Lr3GT* are mainly involved in the glycosylation of carbon 3, and *LrUGT75C1* is related to the glycosylation of carbon 3 and carbon 5. The result suggests that the glycosylated anthocyanin of carbon 3 is necessary for the stable storage of delphinidin in *L. ruthenicum* fruits. Furthermore, previous research has demonstrated that most of the methylation occurs on the *C*3’ and *C*5’ hydroxyl groups of the anthocyanin molecules and sometimes on the *C*5 or *C*7 hydroxyl groups to reduce the chemical activity of the whole molecule and increase its water solubility (Mol et al., 1998). In the fruit of *L. ruthenicum*, *LrAOMT* methylated the delphinidin on its 3’ hydroxyl group to form petunidin or both its 3’ and 5’ hydroxyl groups to form malvidin. Previous experiments have shown that petunidin derivatives accounted for 95% of the total anthocyanins in *L. ruthenicum* fruit, and most of the anthocyanins were acylated by coumaric acid (Zheng et al., 2011). In our study, we identified three transcripts of *Lr3AT* similar to *Gentiana trifloral* belonging to the BAHD acyltransferase family to catalyze anthocyanin acylation (Fujiwara et al., 1998). These results prove that a variety of anthocyanin modifications are found in black fruits but not in white fruits.

### Identiﬁcation of WGCNA Modules Related to Anthocyanin Biosynthesis

WGCNA is now commonly used to analyze entire transcriptome, proteomics, and metabolomics data to identify feature-related co-expression modules (Langfelder and Horvath, 2008). To investigate the gene regulatory network of anthocyanin synthesis in *L. ruthenicum* fruits, a weighted gene co-expression network analysis (WGCNA) was conducted using 23,642 filtered DEGs (**Table S8**). These DEGs identified 35 distinct co-expression modules corresponding to clusters of correlated transcripts (**Figure 4B** and **Table S9**). The total anthocyanin contents of the two kinds of fruits and significant structural genes in transcriptome including *LrCHS*, *LrF3’5’H*, *LrDFR*, *LrANS*, *LrUFGT*, and *Lr3GT* acted as the trait data for a module-trait relationship analysis. The MEdeeppink2 module (4447 genes) presented the highest correlation with anthocyanin (r = 0.98, p = 4e-07) and all structural genes (r<0.05) in the 35 modules (**Figure 4A**). Furthermore, the expression proﬁle of the deeppink2 module indicated that all DEGs were up-regulated with anthocyanin accumulation in black fruits, but almost no expression was detected in white fruits (**Figure 4C**). Based on the analysis above, the deeppink2 modules were selected for further study. The Gene Ontology (GO) enrichment analysis of the “deeppink2” module showed significantly enriched terms associated with binding, protein binding, and catalytic activity (**Figure S7A**). The KEGG pathway enrichment analysis of the “deeppink2” module showed that the first five significant metabolic enrichment pathways were regulation of autophagy, peroxisome, flavonoid biosynthesis, flavone and flavonol biosynthesis, and endocytosis (**Figure S7B**). Cytoscape representation of the genes with WGCNA edge weight >0.25 indicated that these genes were highly positively connected in the “deeppink2” module. In the interaction network diagram, the outer layer consists of 24 hub genes, including phenylpropanoid-biosynthesis genes *PAL*, *BGLU11*; flavonoid synthesis genes *CHS*, *F3H*, *F3’H*, and *F3’5’H*; and anthocyanin synthesis genes *DFR*, *ANS*, *AOMT*, *3GT*, and *3AT* (**Figure 4D**). In the middle of the network diagram, 10 anthocyanin transporter hub genes showed the highest node connectivity with the 24 structural genes (**Figure 4D**). The transcripts of 7 hub genes of transcription factors were identified in the center of the network diagram including *LrMYB3*(Cluster-11506.171206 and Cluster-11506.161621), *LrETC1*(Cluster-11506.49726), *LrMYBX* (Cluster-11506.134566), *LrAN1b* (Cluster-11506.84096 and Cluster-11506.84101), *LrbHLH 128* (Cluster-11506.180271), *LrTTG2* (Cluster-11506.82488), and *LrNAC78* (Cluster-11506.127677 and Cluster-11506.127679), which are orthologous genes in Arabidopsis and petunia (**Figure 4D**). All Hub genes are listed in **Table S10**. These results further illustrate that the deeppink2 module is very relevant to anthocyanin synthesis.

### Conﬁrmation of the Transcriptome Data Using qRT-PCR

**Figure 5:**
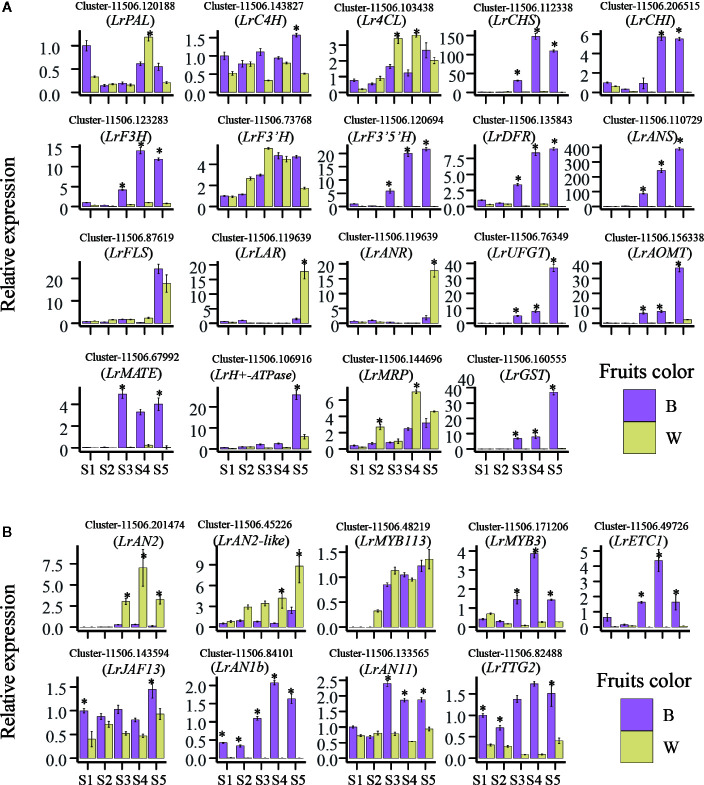
Quantitative real-time polymerase chain reaction (qRT-PCR) verify anthocyanin-related genes. **(A)** Anthocyanin biosynthesis and **(B)** anthocyanin-regulated transcription factor expression at different developmental stages of black and white fruits (S1–S5). Each value is the average of three replicates, and error bars represent SEM (standard error of the mean). Statistical signiﬁcance was determined by the Student’s t-test: *p < 0.05.

To visualize the reliability of the RNA-seq data, 19 genes involved in phenylpropane, flavonoid and anthocyanin synthesis pathways were used for qRT-PCR, and the results were consistent with transcriptome data (**Figure 5A**). At the same time, we screened important 4 Hub genes *LrMYB3*, *LrETC1*, *LrAN1b*, *LrTTG2*, 5 anthocyanin related transcription factors *LrAN2*, *LrMYB113*, *LrJAF13*, *LrAN11* identified from previous study, A newly identified MYBs transcription factor is designed as *LrAN2-like* (Cluster-11506.45226) through transcriptome data related to anthocyanin metabolism were selected to analyze their expression using qRT-PCR (**Figure 5B**). All genes used for qRT-PCR was listed in **Table S11**. The results showed that the expression level of Hub genes gradually increased in black fruits, and then the expression of these genes during the development of white fruits was extremely low. In particular, *LrETC1* and *LrAN1b* were hardly expressed. In addition, the expression levels of *LrJAF13* and *LrAN11* in black fruits were significantly higher than those in white fruits in the later stages of fruit development. In contrast, the expression levels of *LrAN2* and *LrAN2-like* are significantly higher in white fruits than in black fruits, and *LrMYB113* is not different between the two fruits. Among them, *LrAN2-like* has the highest expression level among the three genes. Overall, the qRT-PCR results of all candidate genes are very consistent with the corresponding transcriptional abundance of RNA-Seq (**Figures 3** and **5B**), verifying that the RNA-Seq results expressed by these candidate genes are reliable.

### Sequence Alignment and Phylogenetic Analysis

**Figure 6:**
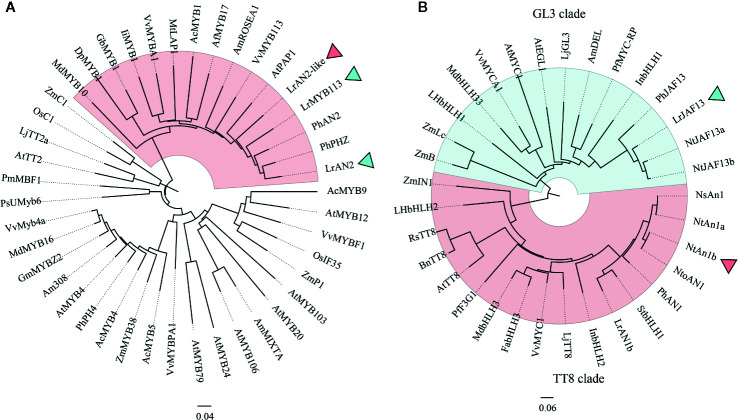
The neighbor-joining phylogenetic tree of plant MYB and bHLH TF sequences. Numbers next to the nodes are bootstrap values from 1,000 replications. The tree is drawn to scale and its branch length is the same as the unit used to infer the evolutionary distance of the phylogenetic tree (scale bar, 0.1 amino acid substitutions per site). The NCBI database retrieves the following deduced amino acid sequences: **(A)** AcMYB1(AQP25672.1), AcMYB4(AQP25673.1), AcMYB5(AQP25674.1), AcMYB9(AQP25675.1) in *Allium cepa*; AmROSEA1(ABB83826.1), AmMIXTA (CAA55725.1) and Am308(P81393.1) in *Antirrhinum majus*; AfMYB17(ACQ82820.1)in *Aquilegia Formosa*; AtMYB4(AAP13410.1), AtMYB12(ABB03913.1), AtMYB20(NP_176797.1), AtMYB24(AAD53092.1), AtPAP1(AAG42001.1), AtMYB79(AEE83284.1), AtMYB103(AED96722.1), AtMYB106(AEE73615.1) and AtTT2(CAC40021.1) in *Arabidopsis thaliana*; DpMYB1 (BAJ33513.1) in *Dahlia pinnata*; GmMYBZ2(NP_001235092.2) in *Glycine max*, GbMYB1(BAJ17661.1) in *Gynura bicolor*; IiMYB1(BAE94391.1) in *Ipomoea nil*; LjTT2a (BAG12893.1) in Lotus japonicus; MdMYB10(ACQ45201.1) and MdMYB16(ADL36756.1) in *Malus domestica*; MtLAP1(ACN79541.1) in *Medicago truncatula*; OsC1(BAD04024.1), OsIF35(BAB64301.1) in *Oryza sativa*; PhAN2 (AAF66727.1), PhPH4(ADX33331.1),PhPHZ(ADW94951.1) in *Petunia x hybrida*; PsUMyb6 (ACH95792.1) in *Phalaenopsis schilleriana*; PmMBF1(AAA82943.1) in *Picea mariana*; VvMYBA1(BAD18977.1),VvMyb4a(ABL61515.1),VvMYBPA1(CAJ90831.1),VvMYBF1(ACT88298.1),VvMYB113(RVW78283.1) in *Vitis vinifera*; ZmC1(AAA33482.1, ZmP1(AAC49394.1), ZmMYB38(P20025.1) *Zea mays*; LrAN2(MK125045.1), LrAN2-like (MT749386), and LrMYB113 (MT773444) *Lycium ruthenicum*. **(B)** AmDEL(AAA32663) in *Antirrhinum majus*, BnTT8 (NP_001302903) in *Brassica napus*,FabHLH3 (AFL02463) in *Fragaria × ananassa*, (BAE20057) and LhbHLH2 (BAE20058) in *Lilium hybrid*,InbHLH1(BAE94393) and InbHLH2(BAE94394) in *Ipomoea nil*; MdbHLH3 (ADL36597) and MdbHLH33 (ABB84474) in *Malus domestica*, PhAN1 (AAG25928), and PhJAF13 (AAC39455) in *Petunia ×hybrida*, VvMYC1 (ACC68685) and VvMYCA1 (ABM92332) in *Vitis vinifera*, ZmB (CAA40544), ZmIN1 (AAB03841), and ZmLc (P13526) in *Zea mays* LjGL3 (AB492284) and LjTT8 (AB490778) in *Lotus japonicus*; NsAN1 (HQ589210) in *Nicotiana sylvestris*; NtAN1a (HQ589208), NtAN1b (HQ589209), NtJAF13a (KF305768), and NtJAF13b (KF298397) in *Nicotiana tabacum*, NtoAN1 (HQ589211) in *Nicotiana tomentosiformis* PfF3G1 (AB103172) and MYC-RP (AB024050) in *Perilla frutescens*, RsTT8 (KY651179) in *Raphanus sativus*;StbHLH1 (ALA13578.1)in *Solanum tuberosum*; AtEGL3 (Q9CAD0), AtGL3 (NP_680372), AtMYC1 (Q8W2F1), and AtTT8 (Q9FT81) in *Arabidopsis thaliana*; LrJAF13(KF768076), LrAN1b(KF768077) in *Lycium ruthenicum*.

Phylogenetic analysis was employed to analysis the anthocyanin-related R2R3-MYB proteins LrAN2 (Cluster-11506.201474), LrAN2-like (Cluster-11506.45226), LrMYB113(Cluster-11506.48219) and bHLH proteins LrAN1b (Cluster-11506.84101) and LrJAF13 (Cluster-11506.143594) in *L. ruthenicum*. At the same time, homologous proteins of other different plants were downloaded to analyze the phylogenetic relationship with these genes (**Figures 6A, B**). Three proteins were identified cluster with anthocyanin regulators PhAN2 and PhPHZ (*Petunia* x *hybrida*), AtPAP1(*Arabidopsis thaliana*) (Borevitz et al., 2000; Albert et al., 2011). Meanwhile, we found that LrAN1b and LrJAF13 were clustered into two clades, namely TT8 and GL3 clades, as previously described (Zhang B. et al., 2019). LrAN1b belongs to the TT8 clade, most homologous with StbHLH (*Solanum tuberosum*) and PhAN1(*Petunia* x *hybrida*), while another LrJAF13 was clustered with NtJAF13a, NtJAF13a (*Nicotiana tabacum*) and PhJAF13 (*Petunia* x *hybrida*) (Quattrocchio et al., 1998). Previous studies show that both branches of bHLH group have the function of regulating anthocyanin, but there is no functional redundancy, and TT8 clade has a more direct role in regulating anthocyanin (Montefiori et al., 2015).

### LrAN2-Like Interact With LrAN1b and the WD40 Protein LrAN11

**Figure 7:**
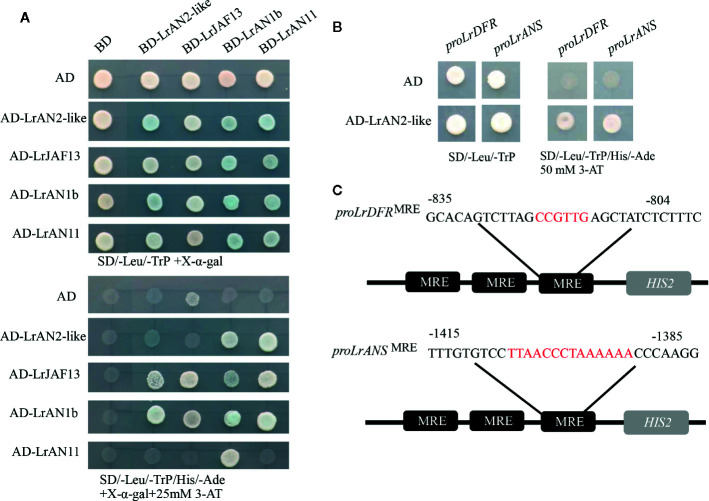
LrAN2-like interacts with other transcription factors and regulates downstream target genes. **(A)** Yeast two-hybrid assay of LrAN2-like and main regulators bHLH protein LrAN1b, LrJAF13 and WD40 protein LrAN11in anthocyanin biosynthesis. The assay shown represents growth on selective media lacking Leu, Ade, Trp, and His, supplemented with 25 mM 3-AT. AD, GAL4 activation domain; BD, GAL4 DNA binding domain. **(B)** Yeast one-hybrid indicates LrAN2-like directly interacts with the MRE in *LrDFR* and *LrANS* promoter. **(C)** The *LrDFR* (-835 to -804) and *LrANS* (-1415 to -1385) promoter sequence containing the MYB-recognizing element (MRE) motif was repeated three times and fused to the *HIS2* reporter gene in Y1H assay.

The MBW complex regulates the expression of genes involved in anthocyanin biosynthesis, analysis of the protein fragments shows that R2R3 MYB and WD40 proteins all bind to different regions of the bHLH protein (Payne et al.). Yeast two-hybrid (Y2H) experiments were performed to determine if LrAN1b could bind other TFs involved in anthocyanin regulation (**Figure 7A**). The molecular interactions candidate gene LrAN2-like were performed with the bHLH proteins LrAN1b and LrJAF13, and the WD40 protein LrAN11 (**Figure 7A**). The result showed that LrAN2-like tested interacted with LrAN1b and LrJAF13, but with varying strengths; LrAN2-like shows a higher affinity for LrAN1b than for LrJAF13. It was observed that the bHLH factors LrAN1b and LrJAF13 not only interacted with themselves, but also that these two transcription factors interacted. In addition, BD-LrAN11 has a strong interaction relationship with all MYBs and bHLHs, which prove WD40 is a crucial component of the MBM protein complex. The Y2H assays contained 25 mM of 3-amino-1,2,4-triazole (3-AT), which inhibits the action of the HIS3 reporter gene to reduce the self-activation of transcription factors. Adding chromogenic substrate X-α-Gal to the selection medium (-Leu-TrP-His-Ade) can activate the LacZ reporter gene.

### LrAN2-Like Proteins Bind to the Promoters of Anthocyanin Biosynthetic Genes

To verify the speculation that anthocyanin biosynthetic genes might be regulated by *LrAN2-like* in *L. ruthenicum*, a yeast one-hybrid (Y1H) assay was employed to explore their ability to bind the promoters of key structural gene *LrDFR* and *LrANS*. Thirty nucleotides from the *LrDFR* (-804 to -835) and *LrANS* (-1385 to -1415) core binding sequence were repeated three times, which were placed upstream of the *HIS3* selectable marker gene (**Figure 7B**). The MRE binding site-*HIS3* construct and full-length LrAN2-like fused to the GAL4 activation domain were co-transformated in Y187. In the selection medium (-Leu-TrP-His-Ade) with 50mM 3AT, LrAN2-like could activate *LrDFR* and *LrANS*, but the negative controls were not (**Figure 7B**). Above all, we deduced that the anthocyanin biosynthetic genes *LrDFR* and *LrANS* might be candidate target genes of LrAN2-like.

### Transient Overexpression of LrAN2-Like and LrAN1b in Tobacco Leaves

**Figure 8:**
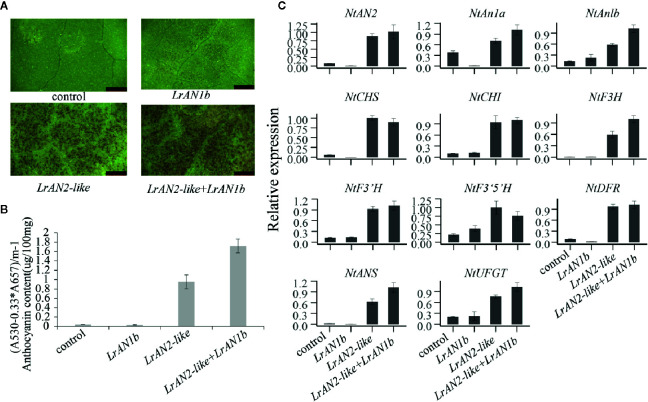
Anthocyanin contents in transiently transformed tobacco leaves infiltrated with Agrobacterium strains carrying *LrAN2-like* and *LrAN1b*. **(A)** Images of transiently transformed tobacco leaves 5 d after agroinfiltration. Four different assays are indicated control: empty vector, *LrAN1b*, *LrAN2-like*, *LrAN2-like*, and *LrAN1b*. **(B)** Anthocyanin contents of four different samples with three biological replicates. **(C)** Relative expression levels of tobacco anthocyanin structural and regulatory genes determined by qPCR analysis. Results represent mean values ± SD from three biological replicates.

To further verify the function of newly identified genes *LrAN2-like* and candidate gene *LrAN1b* in anthocyanin synthesis, we transiently transformed these genes into tobacco leaves. As shown in **Figure 8A**, anthocyanin was not observed when the empty vector pEAQ-HT and *LrAN1b* alone was transformed in tobacco leaves. However, A large amount of pigment accumulated in the tobacco leaves, when *LrAN2-like* was injected into the leaves. This shows that *LrAN2-like* can activate the anthocyanin pathway of tobacco leaves. Moreover, the pigment was largely enhanced when *LrAN2-like* was co-transformed with *LrAN1b*. The anthocyanin content measurement results show that *LrAN2-like* can cause tobacco leaves to produce a large amount of pigment content, and co-transformation of *LrAN1b* can produce the most pigment (**Figure 8B**). To further investigate the mechanism of *LrAN2-like* and *LrAN1b* in regulating anthocyanin metabolism, we evaluated the gene expression of three key anthocyanins regulate transcription factors *NtAN2*, *NtAn1a*, *NtAn1b* and 8 anthocyanin biosynthesis genes *NtCHS*, *NtCHI*, *NtF3H*, *NtF3’H*, *NtF3’5’H*, *NtDFR*, *NtANS*, *NtUFGT* in tobacco by RT-qPCR analysis (**Figure 8C**). As shown in **Figure 8C**, *LrAN2-like* could stimulate the expression of tobacco transcription factors and structural genes, activating anthocyanin synthesis in tobacco leaves. In addition, when *LrAN2-like* and *LrAN1b* were co-expressed, the related genes relative tobacco anthocyanin had a higher upregulation compared to the expression of *LrAN2-like* alone. QRT-PCR analysis suggested that co-transformation of TFs, *LrAN2-like* and *LrAN1b*, enhanced the anthocyanin synthesis and transport by upregulating the expression of the *NtAN2*, *NtAn1a*, *NtAn1b* to activate tobacco anthocyanin synthesis structural. The results indicated that *LrAN2-like* was essential for regulating anthocyanin biosynthesis while *LrAN1b* could facilitate increased anthocyanin accumulation.

### Overexpression of *LrAN1b* Elevated Anthocyanin Accumulation in Florals and Capsules of Transgenic Tobacco

**Figure 9:**
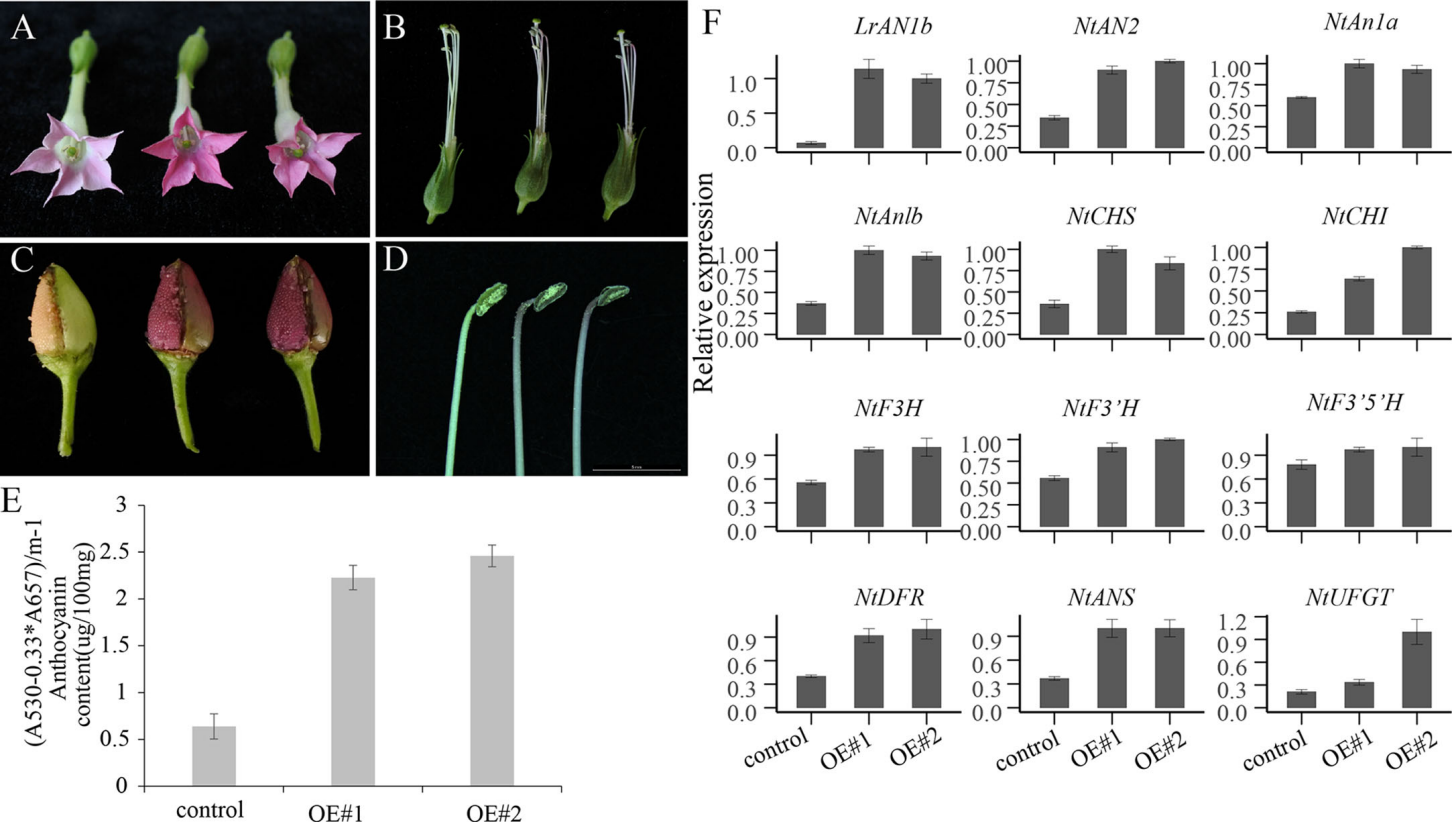
Phenotypic observation and quantitative real-time polymerase chain reaction (qRT-PCR) analyses of the anthocyanin pathway genes in empty vector (control) and two OE-*LrAN1b* line (OE#1, OE#2). the phenotypic images control(left) and OE#1 and OE#2 (right) of **(A)** flower, **(B)** stamen and pistil, **(C)** capsule, **(D)** anther. **(E)** anthocyanin extracted from corollas of the control (left) and OE#1 and OE#2 plants. **(F)** Relative expression of *LrAN1b*, anthocyanin biosynthetic genes, and tobacco transcription factor (TF) genes in the corollas of the control in OE#1 and OE#2 plants. Error bars indicate the standard deviations (SD) of average results. qRT-PCR and anthocyanin determination analyses were performed on three biological replicates.

We generated two T1 over-expressing *LrAN1b* transgenic lines, designed OE#1, OE#2 and used for further analysis (**Figure 9A**). Only increased pigmentation was observed in the florals and capsules of transgenic plants. Anthocyanins in florals tissues accumulate in the corolla, filaments, and anthers (**Figures 9A, B, D**). In addition, in the epidermis of capsules, immature seeds will accumulate large amounts of anthocyanins (**Figure 9C**), but not in other plant tissues of transgenic plants observed. The total anthocyanins content of the transgenic *LrAN1b* OE#1 and OE#2 corollas was 3.6 times and 4.2 times higher than that of the control (**Figure 9E**). We used corollas of two *LrAN1b* overexpression lines OE#1, OE#2 and empty vector control to extract mRNA for qRT-PCR analysis. The results showed that *LrAN1b* of the two lines were significantly increased but not expressed in the control (**Figure 9F**). In addition, qRT-PCR analysis was performed on three key transcription factors *NtAN2*, *NtAn1a*, *NtAn1b* and 8 structural genes involved in anthocyanin synthesis in tobacco anthocyanins pathway. As shown in **Figure 9F**, the transcription levels of *NtAN2*, *NtAn1a*, *NtAn1b* and structural genes *NtCHS*, *NtCHI*, *NtF3H*, *NtF3’H*, *NtF3’5’H*, *NtDFR*, *NtANS*, *NtUFGT* were 2.0–3.0 -fold higher than the control, respectively. In summary, *LrAN1b* is a homologous gene of *NtAn1a* and *NtAn1b* (**Figure 6B**), and overexpression of *LrAN1b* can greatly up-regulate the anthocyanin of tobacco florals and capsules.

## Discussion

### LrAN2-Like Interacts With LrAN1b to Participate in Anthocyanin Regulation

MYB proteins are key factors in regulatory networks controlling secondary metabolism and responses to biotic and abiotic stresses (Dubos et al., 2010). Previous studies identified anthocyanin-activated MYB transcription factor *LrAN2*, *LrMYB113* in *L. ruthenicum* (Zeng et al., 2014). In this study, a new gene MYB transcription factor homologous to *LrAN2* was identified and described as *LrAN2-like*. Recent research indicates that the functional diversity and high expression level of *LrAN2* may be the reason for the high content of anthocyanins in *L. ruthenicum* fruit (Zong et al., 2019). However, the qRT-PCR results indicated that those transcription factors including *LrAN2*, *LrAN2*-like, and *LrMYB113* were still highly expressed in white fruits compared with black fruits (**Figure 5B**). Among them, *LrAN2*-like is the highest expression transcription factor. Sequence alignment and phylogenetic analysis showed that *LrAN2-like* is very homologous to *LrAN2* and *LrMYB113*. Besides, LrAN2-like has extremely high homology to the petunia anthocyanin regulatory gene *PhAN2* (Albert et al., 2011). Y2H indicate that LrAN2-like can interact strongly with LrAN1b and LrAN11, and weakly interact with LrJAF13; indicating that LrAN2-like was able to form MBW complex with bHLH and WD40 proteins. In addition, it was proved that LrAN2-like can directly bind to the MRE of the *LrDFR* and *LrANS* by the Y1H experiment. In order to certify the function of anthocyanin *LrAN2*-like more intuitively, *LrAN2*-like was instantaneously injected into tobacco leaves. It was found that *LrAN2*-like could produce anthocyanins on the leaves in a short time. Several studies indicate that *LrAN2*-like is a new transcription factor regulating anthocyanins different from *LrAN2* and *LrMYB113* in *L. ruthenicum*.

*LrAN1b* was identified by WGCNA and it was associated with anthocyanin accumulation in two fruits and was not expressed in white fruits. The abnormal expression of *LrAN1b* paralleled the lack of anthocyanin accumulation in the white fruit of *L. ruthenicum*. Zeng et al. Showed that *LrAN1b* may be involved in the regulation of anthocyanins through functional prediction and qRT-PCR in *L. ruthenicum*, but there is no direct proof to prove it (Zeng et al., 2014). Therefore, in this study, Y2H and tobacco injection experiments showed that *LrAN1b* can interact strongly with *LrAN2-like* and *LrAN11*. Co-expression of *LrAN1b* and *LrAN2-like* could significantly increase the content and activation of anthocyanin in tobacco leaves Anthocyanin synthesis structural gene. To further verify the gene function of *LrAN1b*, it was stably transformed into tobacco. It was observed that there is significant accumulation of pigment in florals and capsules. In addition, the anthocyanin-regulated transcription factors *NtAN2*, *NtAnla*, *NtAnlb*, and anthocyanin synthesis genes in the corolla of tobacco were up-regulated about 2 times. This is similar to the phenotype of *NtAnla* and *NtAnlb* overexpression in tobacco (Bai et al., 2011). Phylogenetic analysis showed that LrAN1b and LrJAF13 belong to TT8 clade and GL3 clade transcription factors, respectively. In fact, previous studies have proposed *AN1* is directly involved in the activation of biosynthetic genes, and *JAF13* is involved in the regulation of *AN1* transcription in the anthocyanin pathway of Solanaceae plants (Montefiori et al., 2015). The LrAN1b homologous protein PhAN1 in petunia, which interacts with all anthocyanin-regulated MYBs to regulate the anthocyanin pathway (Albert, 2015). PhAN1 mutations will affect pigment synthesis, vacuole pH and seed coat development in petunia (Spelt et al., 2000; Spelt et al., 2002; Zhang S. et al., 2020). In summary, LrAN1b interacts with LrAN2-like and LrAN11 to form a complex to regulate anthocyanins in *L. ruthenicum*. And abnormal expression of *LrAN1b* in white fruits may cause the loss of anthocyanins. This is also the case in other plants, The bHLH transcription factor *RLL1* gene of lettuce loses its function of activating anthocyanin biosynthesis due to a 5 bp deletion in some varieties. (Su et al., 2020). Furthermore, the genome-wide association analysis showed that part of the non-purple rice leaf phenotype was due to an insertion of a 6.5-kb Copia-like retrotransposon found in the 5’UTR (-49-bp) of bHLH transcription factor *OsRb* (Zheng et al., 2019). Above all, these findings imply that the LrAN1b is indispensable for regulation of the anthocyanin pathway in *L. ruthenicum* fruits.

### Identiﬁcation of Anthocyanin Transport and Other Hub Transcription Factors Involved in Anthocyanin Biosynthesis

Anthocyanins are eventually transported to the vacuole storage after being synthesized. In this study, three transcripts of glutathione S-transferase (GST) (Cluster-11506.122999, Cluster-11506.160555, Cluster-11506.160556) were identified homologous to the *TT19* gene in Arabidopsis. Research declares that Arabidopsis *TT19* encodes a GST protein catalyzing glutathione and covalently binding to anthocyanins to form glutathione S-conjugate transports to the vacuolar membrane (Sun et al., 2012). Interestingly, in strawberry, overexpression of *GST* can cause proanthocyanidin biosynthetic enzymes to synthesize anthocyanins at an early developmental stage, leading to early fruit ripening (Gao et al., 2019). Thus, *GST* is not only involved in anthocyanin transport but also a potential candidate gene for color breeding. In addition, three genes were predicted to encode the ABC (ATP-binding cassette) transporter superfamily including D family member 1 (*ABCD1*, Cluster-11506.146202), B family member 27 (*ABCB27*, Cluster-11506.94554) and F family member 1 (*ABCF1*, Cluster-11506.144849). ABCC1 is thought to be a transporter located on the vacuolar membrane responsible for transporting cyanidin 3-*O*-glucoside to the vacuole in grapes (Francisco, 2013). Additionally, three multidrug and toxic compound extrusion (*MATE*) (Cluster-11506.55162, Cluster-11506.67992, Cluster-11506.67991) genes were identified in this module, with extremely high expression in black ripe fruits and low expression in white fruits (**Figure 5A**). In grapes, *MATE* can only transport acylated anthocyanins, but not pelargonidin 3-*O*-glucoside or cyanidin 3-*O*-glucoside, meaning that acylation is necessary for MATE transport (Gomez et al., 2009). Furthermore, three V-type H + -ATPase genes (Cluster-11506.124451, Cluster-11506.128173, Cluster-11506.148228) were identified in the deeppink2 module. Previous research has found that the functions and activities of *MATE* largely depend on different types of H ^+^ - ATPase to provide and maintain the H ^+^ concentration gradients on both sides of the vacuole membrane (Zhao and Dixon, 2010). Above all, three types of mechanisms for anthocyanin transport are found in *L. ruthenicum* fruits: GST-mediated transport, MRP and MATE-mediated transport and membrane vesicle-mediated transport.

In addition to the members of the MBW complex, other transcription factors also indirectly regulate anthocyanin biosynthesis. In this study, a homologous protein of PH3 was identified in WGCNA designed as *LrTTG2*, which was up-regulated in black fruits according to anthocyanin accumulation but exhibits extremely low expression in white fruits (**Figures 4D** and **5B**). PH3 encodes a WRKY protein. It is not only the target gene of AN11-AN1-PH4 complex, but also can be combined with AN11 alone to regulate the vacuolar acidification of petunia (Verweij et al., 2016). Furthermore, two transcripts of *LrNAC78* were detected in this module, and their expression levels were higher in black fruits than in white fruits (**Figure 4D**). LrNAC78 is homologous to *ANAC078* in *Arabidopsis thaliana*, which regulates flavonoid production under light (Morishita et al., 2009). Furthermore, *LrMYBX* and *LrbHLH 128* are homologous to MYB-like protein X in *Solanum lycopersicum* and *StbHLH128* in *Solanum tuberosum*, respectively, encoding a protein with unknown function.

On the other hand, we identified a putative R2R3-MYB TF *LrMYB3* and another R3 TF MYB transcription factor *LrETC1* in the center of the network. These two genes were predicted to be negative regulators of anthocyanin biosynthesis (**Figure 4D**). The transcription of *LrMYB3* and *LrETC1* was highly correlated with that of *LrAN1b* and with the degree of fruit coloration. The gene function prediction results showed that *LrMYB3* was most homologous to the petunia transcription factor *PhMYB27*. PhMYB27 contains a C-terminal EAR motif to bind MBW complexes and targets the anthocyanin pathway genes, and its expression increases under light induction to inhibit excessive anthocyanin accumulation and prevent damage to plants (Albert et al., 2014). Additionally, *LrETC1* is very similar to Arabidopsis CAPRICE (*CPC*), TRIPTYCHON (*TRY*), and ENHANCER OF TRY and CPC 1 (*ETC1*). Although these R3MYBs do not have DNA-binding domains, a previous study indicated that *CPC* retains the motifs responsible for binding to bHLH proteins to participate in inhibiting anthocyanin synthesis under different stress conditions (HF et al., 2009). What emerges from the results reported here is that *LrMYB3* and *LrETC1* play a repressor role in the regulation of anthocyanin biosynthesis in *L. ruthenicum*. The specific regulatory mechanism of these two genes is under study, and more work is needed to prove it.

### Research on Anthocyanin Regulation Network *L. ruthenicum* Fruits

In our research, the black mature fruits mainly accumulated delphinidin derivatives while flavonols were detected in white mature fruits to be 2–3 times higher than in black fruits (**Figure 2**). The metabolic difference of the white fruit phenotype is due to the drastic reduction of anthocyanins, and the metabolic flow shifts to the flavonol products. Meanwhile, a large number of anthocyanin metabolism synthesis genes, modified genes, transport genes and transcription factors were identified as candidate genes for pigment changes in two fruit by RNA-seq and WGCNA analysis.

**Figure 10:**
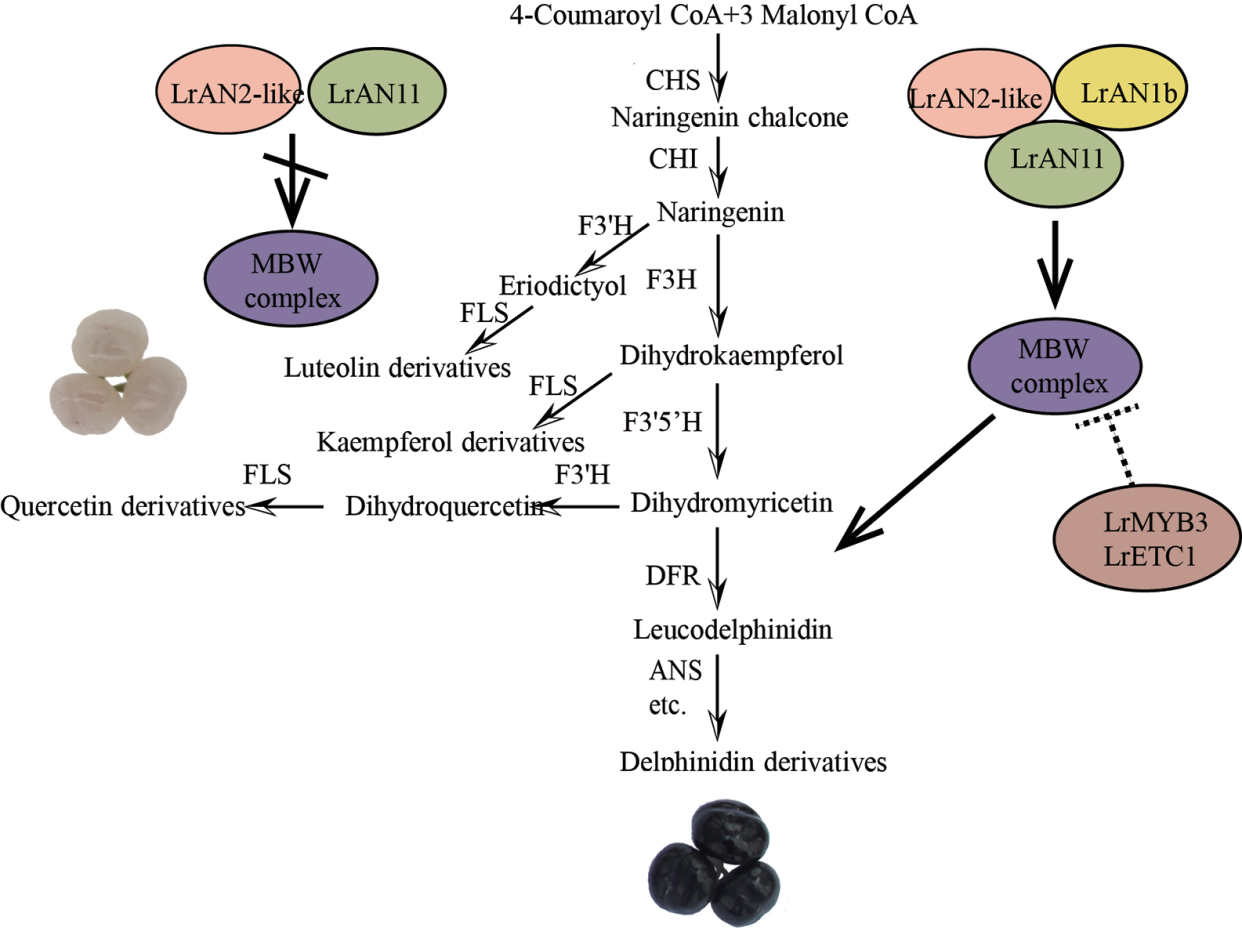
A working model explains the reasons for the specific accumulation of anthocyanins and flavonols in black and white fruits, respectively.

Through gene function verification, we proposed a new working model that explains the specific accumulation of anthocyanins and flavonols in black and white fruits on a metabolic and molecular level (**Figure 10**). In black fruits, LrAN2-like interacts with LrAN1b and LrAN11 to form an MBW complex, which regulates the downstream target genes *LrDFR* and *LrANS* promoters to regulate anthocyanin synthesis. When the accumulation of anthocyanins is too high in the later stages of fruit development, the MBW activation complex activates the genes of the *LrMYB3* and *LrETC1* repressors to achieve feedback inhibition. On the other hand, however, in white fruits, the expression of *LrAN1b* is abnormal, so this dynamic balance mechanism is broken. The MBW complex that depends on *LrAN1b* cannot effectively activate its target genes, especially the key genes *LrDFR* and *LrANS*. Anthocyanin metabolism and flavonols are important branches of flavonoid metabolism. The decrease in *LrDFR* expression leads to the substrate leading to the flavonol branch, as a result, the proportion of flavonols in white is higher than that in black fruits. In other words, *LrMYB3* and *LrETC1* cannot be effectively activated, resulting in the failure of the feedback regulation mechanism during fruit pigmentation. Therefore, abnormal expression of *LrAN1b* will destroy the flavonoid homeostasis network, and the content and proportion of flavonol and anthocyanin in white fruits will be different (**Figure 10**). A similar result was also reported in the white flesh of radish, which was positively correlated with low or undetectable *RsbHLH4* expression (Lai et al., 2020). Another latest study shows abnormal *bHLH3* expression disrupts the homeostatic regulatory network, causing differences in pigment composition among mulberry fruits (Li et al., 2020). Anthocyanin regulation is a complex and variable process. Changes in the synthesis of a gene and transcription factor will affect the composition of the tissue (Paauw et al., 2019). Therefore, the regulation of anthocyanin metabolism of *L. ruthenicum* is worth further investigation and analysis. In this study, our results initially revealed the reasons for the different color of *L. ruthenicum* fruit, and laid the foundation for the analysis of the anthocyanin regulatory network of *L. ruthenicum*.

## Data Availability Statement

The datasets presented in this study can be found in online repositories. The names of the repository/repositories and accession number(s) can be found in the article **Supplementary Material**.

## Author Contributions

LT and YC conceived and designed the study. TL and YF performed the experiment, analyzed the data, and drafted the article. LT and YC reviewed and edited the manuscript. DL, YL, YY, and XQ contributed to data acquisition. HQ, GL, FC, and JW participated in the data analysis and interpretation. All authors contributed to the article and approved the submitted version.

## Funding

This work was financially supported by the Agro-Technical Independent Innovation Special Project of Ningxia Hui Autonomous Region, China (QCYL-2018-04,YES-16-0403), Foreign cooperation project of ningxia academy of agricultural and forestry sciences (DW-X-2018011) and the National Natural Science Foundation of China (31260351).

## Conflict of Interest

The authors declare that the research was conducted in the absence of any commercial or financial relationships that could be construed as a potential conflict of interest.
