# Supplementary material for: Transcriptome and Flavonoids Metabolomic Analysis Identifies Regulatory Networks and Hub Genes in Black and White Fruits of Lycium ruthenicum Murray
Source: Front Plant Sci. 2020 Aug 14;11:1256. doi: 10.3389/fpls.2020.01256 (PMC7456873; doi:10.3389/fpls.2020.01256)
Supplement: Supplementary file 1 [file DataSheet_1.zip › Supplementary Material/Supplementary figure.docx]

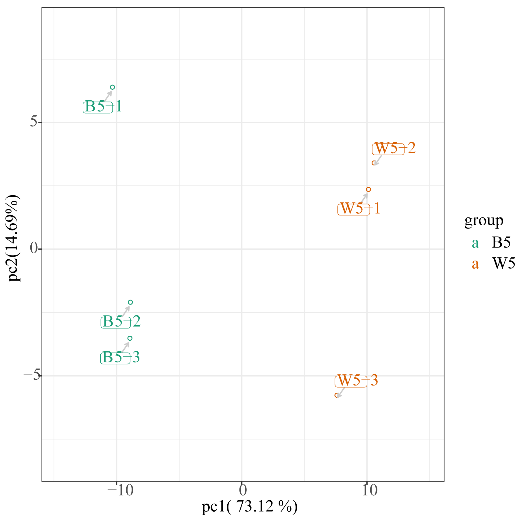


**Supplementary Figure 1.** PCA score plot of metabolite profiles from the B5 and W5 fruits. Each point represents an independent biological replicate.


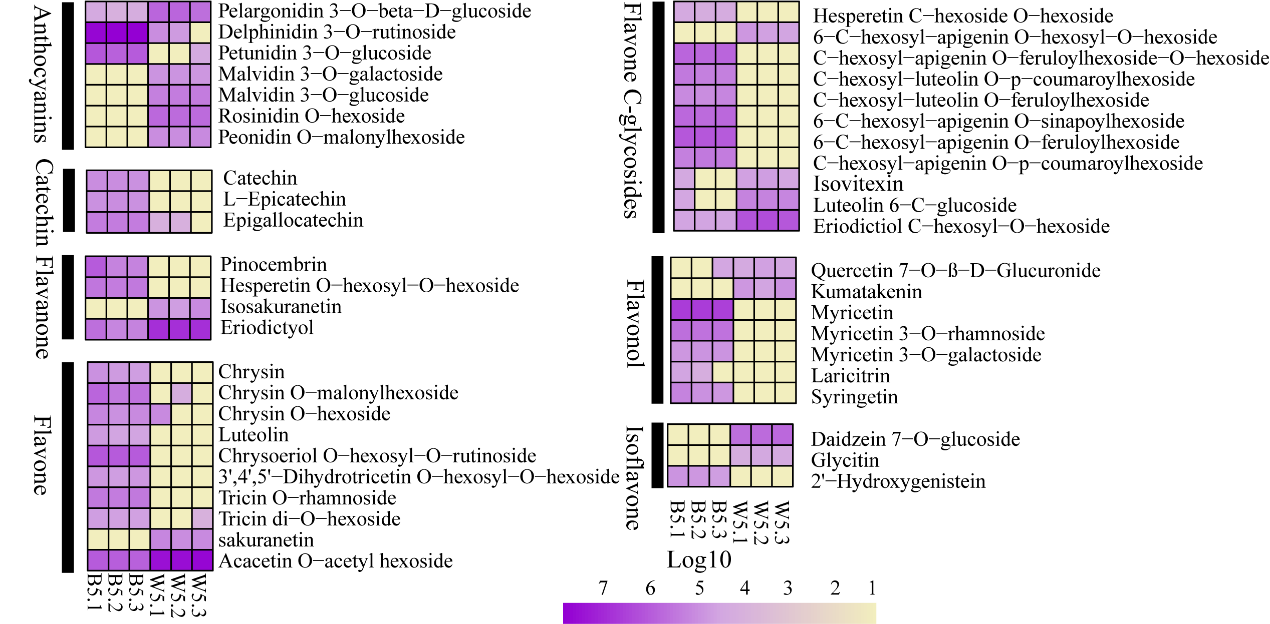


**Supplementary Figure 2.** Heat map of differentially accumulated flavonoid metabolites in two fruits (|Log2 (fold change)| ≥2, p-value < 0.05, and VIP≥1).

A


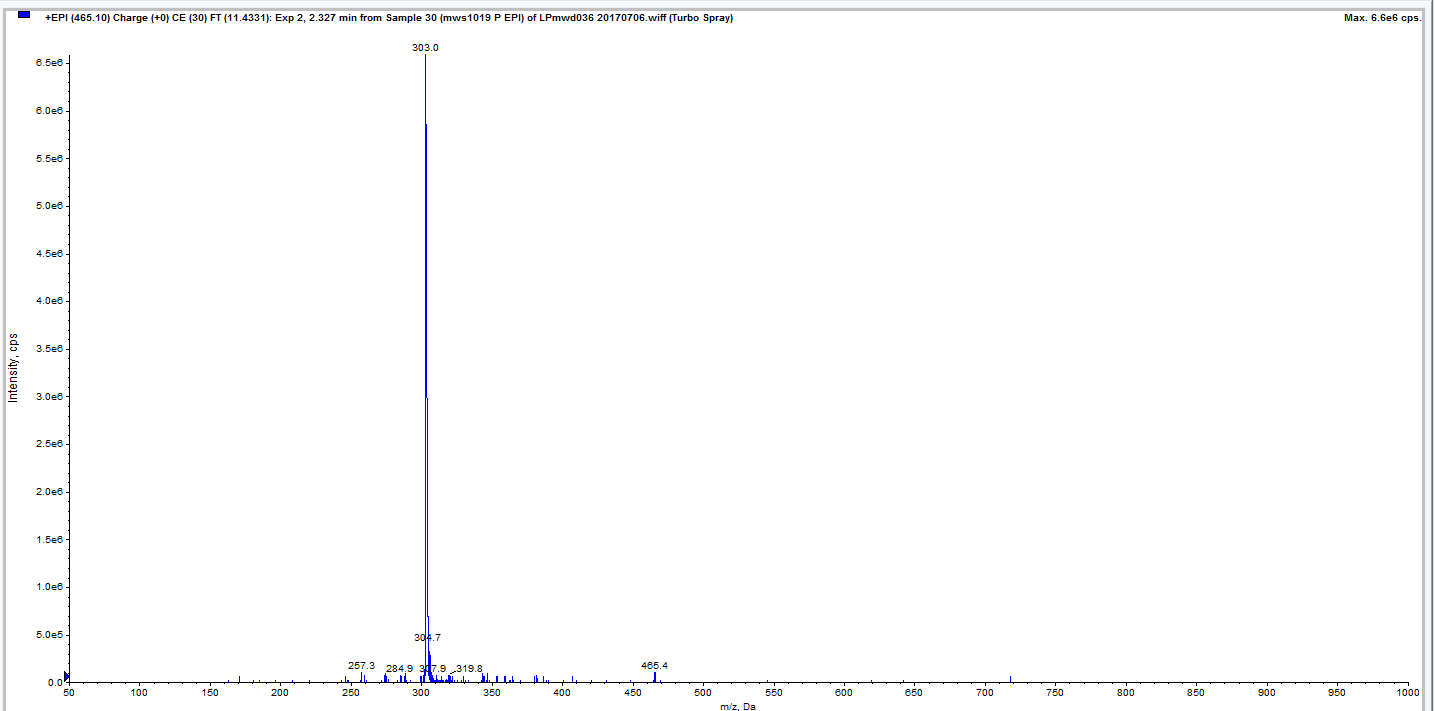


B


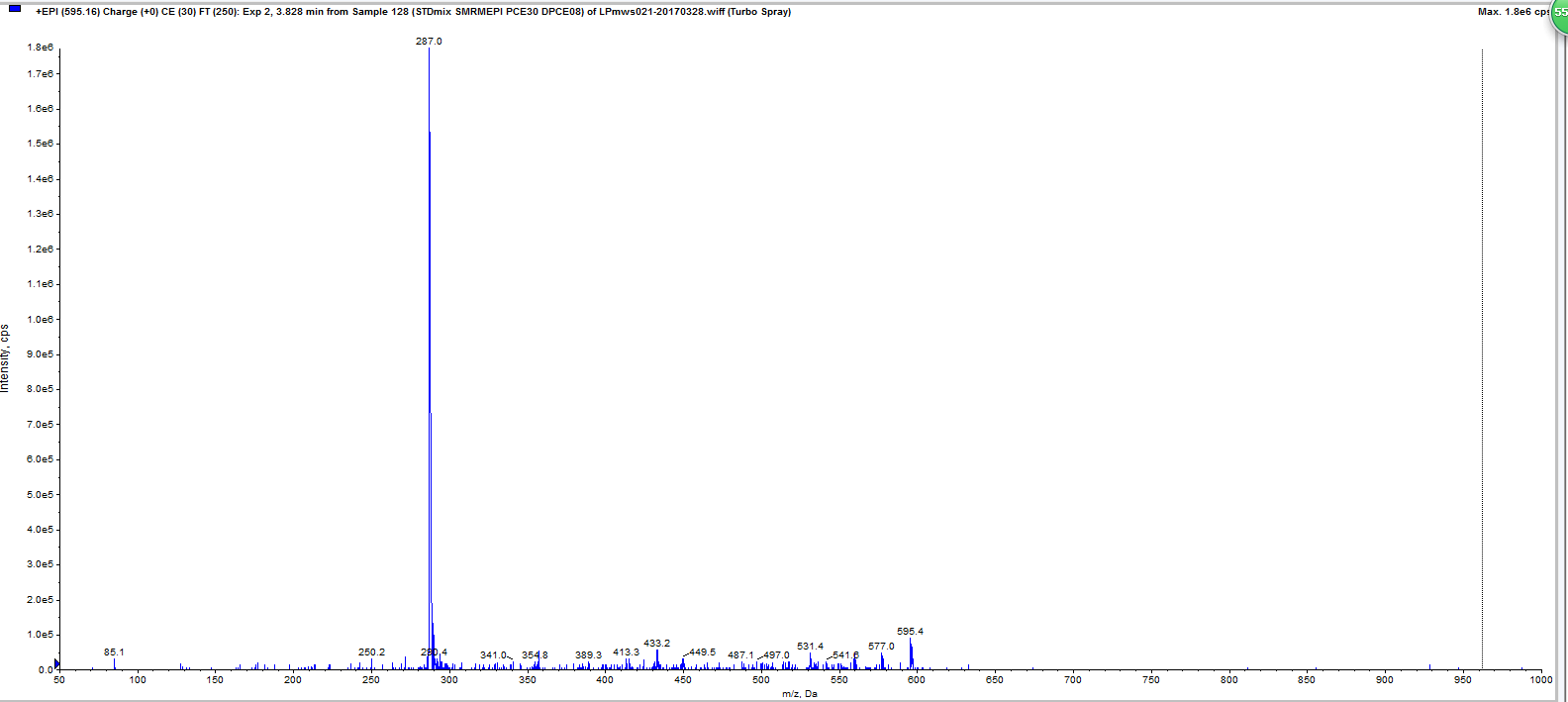


C


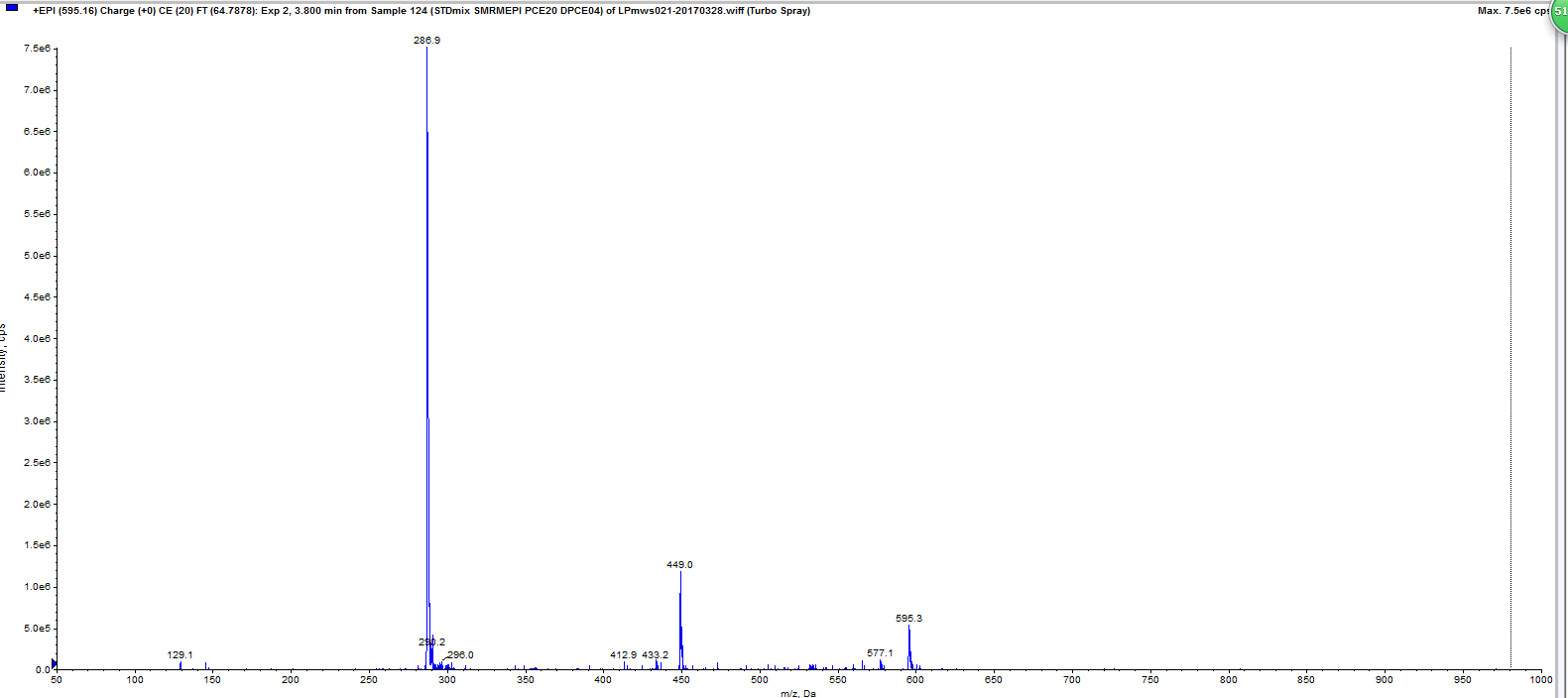


D


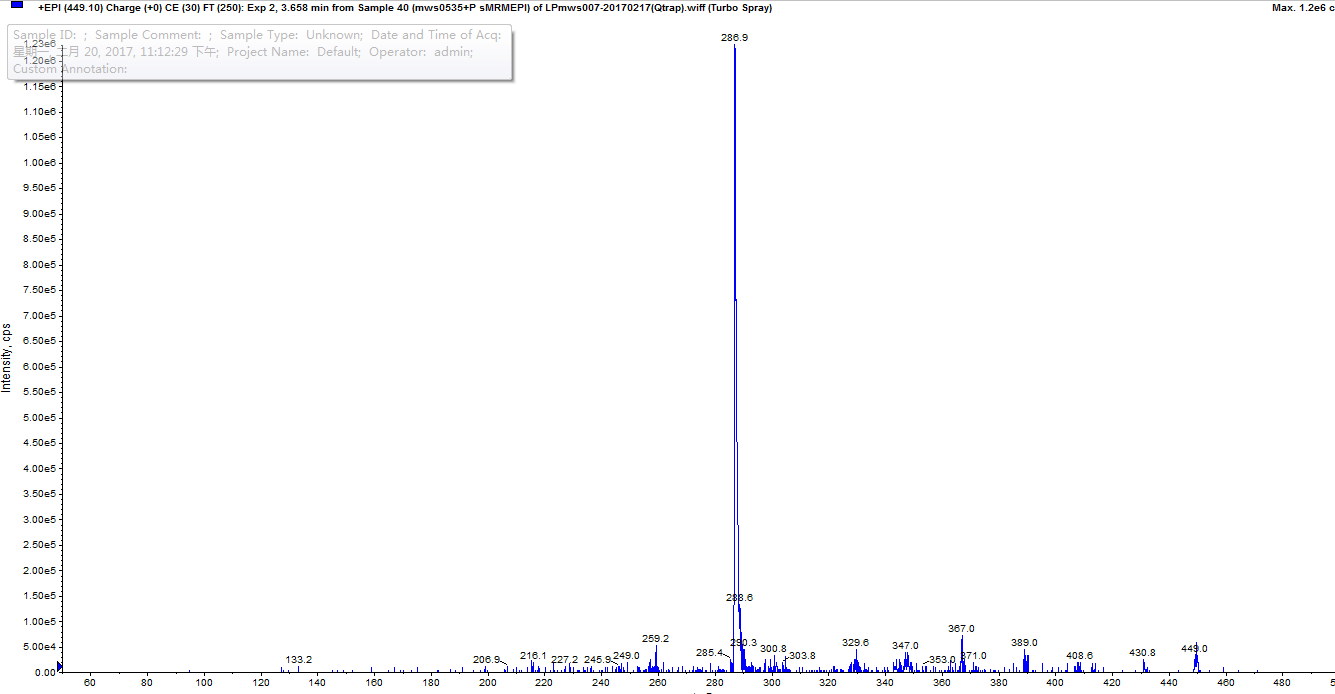


E


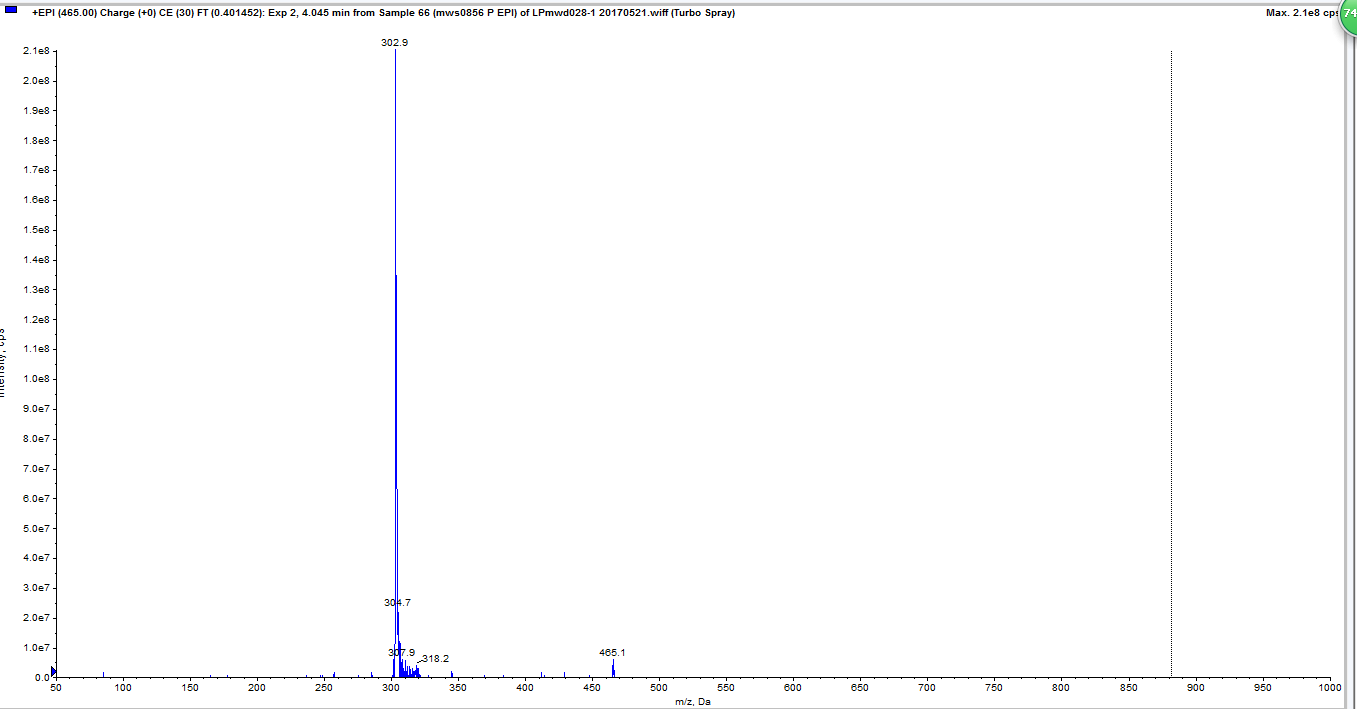


**Supplementary Figure 3.** The secondary mass spectrogram of the main metabolites of the black fruit in *L. ruthenicum* (A) Delphinidin 3-*O*-glucoside, And the main metabolites in the white fruit (B) Kaempferol 3-*O*-rutinoside (C) Kaempferol 3-*O*-robinobioside (D) Luteolin 7-*O*-glucoside (E) Quercetin 4'-*O*-glucoside.

**Supplementary Figure 4.** The analysis of transcript splicing results. (A) frequency distribution map of unigene length of spliced transcripts. (B) frequency distribution map of spliced transcripts and unigene length distribution.


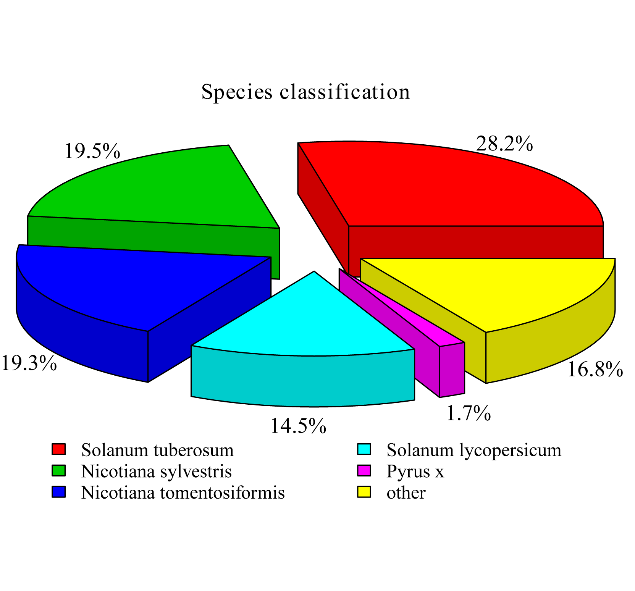


**Supplementary Figure 5.** Species classification of *L. ruthenicum* unigene 3D pie chart

**Supplementary Figure 6.** Statistics of differentially expressed genes (DEG). (A) venn diagram of differentially expressed genes. The sum of the numbers in each large circle represents the total number of differentially expressed genes in the compared combinations, while the overlapping portions of the circles represent the differentially expressed genes that are common between the combinations. (B) cluster heat map analysis of DEG up-regulated by B1vsw1_B2vsw2_B3vsw3_B4vsw4_B5vsw5, red means up-regulation, blue means down-regulation.

**Supplementary Figure 7.** GO (A) and KEGG (B) enrichment analysis for module deeppink2 genes.
