# Supplementary material for: Transcriptome and Flavonoids Metabolomic Analysis Identifies Regulatory Networks and Hub Genes in Black and White Fruits of Lycium ruthenicum Murray
Source: Front Plant Sci. 2020 Aug 14;11:1256. doi: 10.3389/fpls.2020.01256 (PMC7456873; doi:10.3389/fpls.2020.01256)
Supplement: Supplementary file 1 [file DataSheet_1.zip › Supplementary Material/Supplementary tables.docx]

**Supplementary Table S1**

Table S1 Primers used in this study

| Purpose | Primer name | Primer sequence (5′→3′) |
| --- | --- | --- |
|  | LrAN2-like-pGADT7-F- *Eco*RⅠ | GGCCATGGAGGCCAGTGAATTCATGAACAGTACTACTCCTATCT |
|  | LrAN2-like-pGADT7-R- *Bam*HⅠ | GCAGCTCGAGCTCGATGGATCCCTAATTAAGTAGACTCCATAT |
|  | LrJAF13-pGADT7-F- *Eco*RⅠ | GGCCATGGAGGCCAGTGAATTCATGGCTATAGGACACCAAGAT |
|  | LrJAF13-pGADT7-R- *Bam*HⅠ | GCAGCTCGAGCTCGATGGATCCTCAAGATTTCCAGACTACTC |
|  | LrAN1b-pGADT7-F- *Eco*RⅠ | GGCCATGGAGGCCAGTGAATTCATGGCGATCATACAGACTAGC |
|  | LrAN1b-pGADT7-R- *Bam*HⅠ | GCAGCTCGAGCTCGATGGATCCTTAAACTCTAGGGATTATC |
|  | LrAN11-pGADT7-F- *Eco*RⅠ | GGCCATGGAGGCCAGTGAATTCATGGAGAATTCAAGTCAAGAAT |
| Yeast two hybrid | LrAN11-pGADT7-R- *Bam*HⅠ | GCAGCTCGAGCTCGATGGATCCTTATACTTTAAGCATCTGCAAC |
|  | LrAN2-like-pGBKT7-F- *Eco*RⅠ | TATGGCCATGGAGGCCGAATTCATGAACAGTACTACTCCTATC |
|  | LrAN2-like-pGBKT7-R- *Bam*HⅠ | GCCGCTGCAGGTCGACGGATCCCTAATTAAGTAGACTCCATA |
|  | LrJAF13-pGBKT7-F- *Eco*RⅠ | TATGGCCATGGAGGCCGAATTCATGGCTATAGGACACCAAGAT |
|  | LrJAF13-pGBKT7-R- *Bam*HⅠ | GCCGCTGCAGGTCGACGGATCCTCAAGATTTCCAGACTACTC |
|  | LrAN1b-pGBKT7-F- *Eco*RⅠ | TATGGCCATGGAGGCCGAATTCATGGCGATCATACAGACTAGC |
|  | LrAN1b-pGBKT7-R- *Bam*HⅠ | GCCGCTGCAGGTCGACGGATCCTTAAACTCTAGGGATTATC |
|  | LrAN11-pGBKT7-F- *Eco*RⅠ | TATGGCCATGGAGGCCGAATTCATGGAGAATTCAAGTCAAGAAT |
|  | LrAN11-pGBKT7-R- *Bam*HⅠ | GCCGCTGCAGGTCGACGGATCCTTATACTTTAAGCATCTGCAAC |
| Yeast one hybrid | ProLrDFR-pHis2-F- *Eco*RⅠ | GCCATTCAAGGCTGTCAAGGGACTCACTATAGGGCGAATTC |
|  | ProLrDFR-pHis2-R- *Mlu* I | CGGATCGATTCGCGAACGCGTCTAGATTTCACCATTGGTTAAC |
|  | ProLrANS-pHis2-F- *Eco*RⅠ | GACTCACTATAGGGCGAATTCATGGTGAGTGCAGTTGTTCC |
|  | ProLrANS-pHis2-R- *Mlu* I | CGGATCGATTCGCGAACGCGTAGGCCAGATGGCCAAGTCG |
|  | Lractin-RT-F | CTCAGCACCTTCCAGCAGAT |
|  | Lractin-RT-R | TAACACTGCAACCGCATTTC |
| qRT-PCR assay | LrPAL-RT-F | TGATCTTGTCCCTTTGTCC |
|  | LrPAL-RT-R | CCTTCTTTAGGCTGCAACTC |
|  | LrC4H-RT-F | TAGTCAACCACCCTCACATC |
|  | LrC4H-RT-R | AGAGTCTCCTTGACCACAGC |
|  | Lr4CL-RT-F | AAGCAACCTCAGCAACACT |
|  | Lr4CL-RT-R | GGACCTGATATGCCTTGTA |
|  | LrCHS-RT-F | TGGAGGAGTATCGTAAGGC |
|  | LrCHS-RT-R | CTTGTGCTCGCTATCAGTG |
|  | LrCHI-RT-F | GGGAGCTGAGCTAACTGAA |
|  | LrCHI-RT-R | CTAAACGATCACGTATGGC |
|  | LrF3H-RT-F | CGTCAACACTAACGGCTCTA |
|  | LrF3H-RT-R | TTCACCTCTTCTTCCATCAA |
|  | LrF3’H-RT-F | AACATGGAGGAAGCATTTGG |
|  | LrF3’H-RT-R | TGGCCAGATGTGTTAAAAGC |
|  | LrF3’5’H-RT-F | AAGCACAACAAGAAATGGAC |
|  | LrF3’5’H-RT-R | TTTAATGGTGTTGAAGGGTG |
|  | LrDFR-RT-F | TCTGTTAGAATTGCCGAAAG |
|  | LrDFR-RT-R | CTCGAAATCCATAGGTGTTG |
|  | LrANS-RT-F | GCAAATAGTGCTTCTGGTCA |
|  | LrANS-RT-R | CTTTGTTGCTAGGTTCCTTA |
|  | LrFLS-RT-F | CCGTCAGACCATGAAGTTGG |
|  | LrFLS-RT-R | CAAAGCATAATACTGATGAAGTCAC |
|  | LrLAR-RT-F | AAGTGTGGTTGCAGCTTTGACA |
|  | LrLAR-RT-R | ATTTTGGGCATGTCATCCATCT |
|  | LrANR-RT-F | CCTCTCTCATGGCTGGTCCTTA |
|  | LrANR-RT-R | GGGCACGACAAACATCCTCTAC |
|  | LrUFGT-RT-F | AGTCCATGAAAGAGGCAGAG |
|  | LrUFGT-RT-R | AGAACAAGAACCAGCAGTCC |
|  | LrAOMT-RT-F | GGCCTCATTCGACAAGTATA |
|  | LrAOMT-RT-R | AGCAAGAGCAGTAGCCAGTA |
|  | LrMATE-RT-F | TAAGGATGGGTGGAAAGGAT |
|  | LrMATE-RT-R | TGTTAAGATGGCCAACAAGG |
|  | LrH+-ATPase-RT-F | TGCAGCAGCTGCGCTGATGG |
|  | LrH+-ATPase-RT-R | TGAGCGGATCACCCTCGAGA |
|  | LrMRP-RT-F | GGCAGAGTGGTTCTGGTAAA |
|  | LrMRP-RT-R | GAGTGCTGGTTCTTGTTGGA |
|  | LrGST-RT-F | AGCAGGCCTTCTTGGAAAA |
|  | LrGST-RT-R | AATATCCACAACGCTCGTGA |
|  | LrAN2-RT-F | ACTGAGGAAGAAGCAGAAGC |
|  | LrAN2-RT-R | CAATATCAGCGGAAAAGTCA |
|  | LrMYB113-RT-F | ATGAGTACTTCTAATGATAAGC |
|  | LrMYB113-RT-R | CCACCTTAGCCTGCAACTC |
|  | LrMYB3-RT-F | CTAAACATGGTGAAGGTTGCTG |
|  | LrMYB3-RT-R | CAGTTCTCCTCGGTAATCTTCC |
|  | LrETC1-RT-F | GACCGTTCAAGCACATCAGATA |
|  | LrETC1-RT-R | GGCTGGTAGAGTGTCTTGAGTT |
|  | LrJAF13-RT-F | AAGTGATGCTTATCCAGTCG |
|  | LrJAF13-RT-R | TTTGAGCTTGTGAAGTTTCC |
|  | LrAN1b-RT-F | ATGGCGATCATACAGACTAG |
|  | LrAN1b-RT-R | CTCGCCGGCTGATTTGACTC |
|  | LrAN11-RT-F | CTGCGACACGTGATTGGAT |
|  | LrAN11-RT-R | CCAAGCTTTAACCCCTTTCC |
|  | LrTTG2-RT-F | CAGAATGATGTTGGGTTGAC |
|  | LrTTG2-RT-R | CCTGCTCCATTGTGTAGGCC |
|  | NtGPDH-RT-F | CTGGTGCTGATTTCGTTGTG |
|  | NtGPDH-RT-R | TTCGGGCTTGTATTCCTTCTC |
|  | NtCHS-RT-F | TGACACCCACTTGGATAGTTTAG |
|  | NtCHS-RT-R | CGACCTCTGGAATTGGATCAG |
|  | NtCHI-RT-F | CTTTTCTCGCCGCTAAATG |
|  | NtCHI-RT-R | TTTCTGCCACCTTCTCTG |
|  | NtF3H-RT-F | CAAGGCATGTGTGGATATGG |
|  | NtF3H-RT-R | TGTGTCGTTTCAGTCCAAGG |
|  | NtF3′H-RT-F | AGGCTCAACACTTCTCGT |
|  | NtF3′H-RT-R | CATCAACTTTGGGCTTCT |
|  | NtF3′5′H-RT-F | CGCACTACCATACTTAGGAGCCAT |
|  | NtF3′5′H-RT-R | CAGCATCAGGAGTAGAAGCAACAG |
|  | NtDFR-RT-F | AACCAACAGTCAGGGGAATG |
|  | NtDFR-RT-R | TTGGACATCGACAGTTCCAG |
|  | NtANS-RT-F | TGGCGTTGAAGCTCATACTG |
|  | NtANS-RT-R | GGAATTAGGCACACACTTTGC |
|  | NtUFGT-RT-F | GAGTGCATTGGATGCCTTTT |
|  | NtUFGT-RT-R | CCAGCTCCATTAGGTCCTTG |
|  | NtAN2-RT-F | GAAGAAAGGTGCATGGACTG |
|  | NtAN2-RT-R | TCTGCAGCTCTTTCTGCATC |
|  | NtAn1a-RT-F | ACCATTCTCGAACACCGAAG |
|  | NtAn1a-RT-R | TGCTAGGGCACAATGTGAAG |
|  | NtAn1b-RT-F | CTTGAACACTTCTCAAACCGA |
|  | NtAn1b-RT-R | TGCTAGGGCACAATGTGAAG |
|  | LrAN2-like-pEAQ-F- *Smal*Ⅰ | CACCATCACCATCATCCCGGGATGAACAGTACTACTCCTAT |
| Tobacco transient transformation | LrAN2-like-pEAQ-R- *Stu*Ⅰ | TGAAACCAGAGTTAAAGGCCTCTAATTAAGTAGACTCCATA |
|  | LrAN1b-pEAQ-F- *Smal*Ⅰ | CACCATCACCATCATCCCGGGATGATGGCGATCATACAGAC |
|  | LrAN1b-pEAQ-R- *Stu*Ⅰ | TGAAACCAGAGTTAAAGGCCTTTAAACTCTAGGGATTATCC |
|  | LrAN1b-pCM1307-F-*Xbal*Ⅰ | ACCGTCGACGAGCTCTCTAGAATGATGGCGATCATACAG |
| Tobacco transformation | LrAN1b-pCM1307-R-*Kpn*Ⅰ | TTTTGCGGAGTACCCGGGTACCTTAAACTCTAGGGATTATCCGA |

**Supplementary Table S3**

Type and content of flavonoids in two kinds fruit of *L. ruthenicum*

| Index | Compounds | Peak Area |  |  |  |
| --- | --- | --- | --- | --- | --- |
|  |  | B5 | SE | W5 | SE |
| pmb0835 | Gallocatechin-gallocatechin | 2.88E+04 | 1.62E+03 | 1.79E+05 | 2.50E+04 |
| pmb2831 | Protocatechuic acid O-glucoside | 3.20E+06 | 9.74E+04 | 7.73E+06 | 1.97E+05 |
| pme0205 | Catechin | 8.46E+04 | 3.54E+03 | 9.00E+00 | 0.00E+00 |
| pme0450 | L-Epicatechin | 7.71E+04 | 3.05E+03 | 9.00E+00 | 0.00E+00 |
| pme1486 | Epigallate catechin gallate | 1.11E+03 | 9.01E+02 | 9.00E+00 | 0.00E+00 |
| pme1516 | Epigallocatechin | 1.94E+05 | 4.53E+03 | 6.24E+03 | 2.55E+03 |
| pme1824 | Protocatechuic acid | 8.13E+05 | 2.38E+04 | 5.64E+05 | 6.49E+04 |
| pme2478 | Protocatechuic aldehyde | 3.86E+04 | 3.99E+03 | 1.04E+05 | 1.01E+04 |
| pmb0541 | Cyanidin 3-O-glucosyl-malonylglucoside | 1.84E+04 | 3.02E+03 | 1.66E+04 | 5.66E+02 |
| pmb0545 | Rosinidin O-hexoside | 9.00E+00 | 0.00E+00 | 4.44E+05 | 1.21E+04 |
| pmb0550 | Cyanidin 3-O-glucoside | 7.33E+04 | 2.95E+03 | 4.12E+05 | 5.47E+04 |
| pmb2957 | Cyanidin O-syringic acid | 1.63E+04 | 1.91E+03 | 2.66E+04 | 1.52E+03 |
| pmb2961 | Peonidin O-malonylhexoside | 9.00E+00 | 0.00E+00 | 8.91E+04 | 3.75E+03 |
| pme0442 | Delphinidin | 8.93E+04 | 1.34E+04 | 1.29E+06 | 1.65E+05 |
| pme0443 | Malvidin 3-O-galactoside | 9.00E+00 | 0.00E+00 | 6.14E+04 | 1.91E+03 |
| pme0444 | Malvidin 3-O-glucoside | 9.00E+00 | 0.00E+00 | 1.82E+05 | 7.66E+03 |
| pme1398 | Delphinidin 3-O-glucoside | 1.04E+07 | 1.17E+06 | 4.56E+06 | 6.08E+04 |
| pme1773 | Cyanidin 3-O-rutinoside | 2.29E+06 | 5.06E+05 | 8.71E+04 | 1.00E+04 |
| pme1777 | "Cyanidin 3,5-O-diglucoside | 4.51E+04 | 6.57E+03 | 8.54E+03 | 3.62E+03 |
| pme3256 | Delphinidin 3-O-rutinoside | 6.51E+07 | 5.62E+06 | 4.45E+04 | 2.05E+04 |
| pme3391 | Petunidin 3-O-glucoside | 8.83E+05 | 1.02E+05 | 6.27E+03 | 5.11E+03 |
| pme3392 | Pelargonidin 3-O-beta-D-glucoside | 1.28E+04 | 2.00E+03 | 5.19E+05 | 6.65E+04 |
| pme3609 | Cyanidin | 1.52E+04 | 9.84E+02 | 1.81E+05 | 1.03E+04 |
| pma0249 | Selgin 5-O-hexoside | 3.11E+06 | 2.37E+05 | 5.00E+05 | 7.28E+04 |
| pma0294 | Chrysoeriol 5-O-hexoside | 2.03E+05 | 3.61E+04 | 1.41E+06 | 1.85E+05 |
| pma0760 | Selgin O-malonylhexoside | 4.62E+04 | 9.27E+02 | 6.81E+04 | 4.95E+03 |
| pma0795 | Tricetin O-malonylhexoside | 1.76E+05 | 2.11E+04 | 1.91E+06 | 2.37E+05 |
| pma0825 | Chrysin O-malonylhexoside | 3.65E+05 | 8.17E+04 | 4.27E+03 | 3.48E+03 |
| pma3443 | Tricin 7-O-acetylglucoside | 1.64E+05 | 1.58E+04 | 6.43E+04 | 8.78E+03 |
| pma6199 | Chrysin O-hexoside | 9.47E+04 | 8.39E+03 | 3.57E+04 | 2.91E+04 |
| pma6373 | "3',4',5'-Dihydrotricetin O-hexosyl-O-hexoside" | 4.76E+04 | 2.54E+03 | 9.00E+00 | 0.00E+00 |
| pma6576 | Spinacetin | 2.32E+05 | 2.37E+04 | 2.96E+04 | 2.45E+03 |
| pmb0277 | Tricin | 1.48E+06 | 9.98E+04 | 1.32E+05 | 1.14E+04 |
| pmb0569 | Syringetin 5-O-hexoside | 7.07E+06 | 7.78E+05 | 6.58E+05 | 1.68E+04 |
| pmb0580 | Chrysin 5-O-glucoside | 1.37E+05 | 1.37E+04 | 5.57E+04 | 1.53E+04 |
| pmb0588 | "Luteolin 3',7-di-O-glucoside" | 2.36E+04 | 1.05E+03 | 4.93E+04 | 3.70E+03 |
| pmb0592 | Chrysoeriol O-hexosyl-O-rutinoside | 9.96E+05 | 4.79E+04 | 9.00E+00 | 0.00E+00 |
| pmb0602 | Syringetin 7-O-hexoside | 6.21E+06 | 4.99E+05 | 5.56E+05 | 1.60E+04 |
| pmb0603 | Chrysoeriol O-hexosyl-O-hexoside | 1.41E+05 | 1.66E+04 | 1.98E+05 | 2.40E+04 |
| pmb0607 | Chrysoeriol 7-O-hexoside | 3.12E+05 | 3.18E+04 | 1.76E+06 | 2.62E+05 |
| pmb0608 | Chrysoeriol O-malonylhexoside | 9.47E+04 | 7.92E+03 | 1.77E+05 | 2.19E+04 |
| pmb0712 | Tricin 5-O-hexosyl-O-hexoside | 1.29E+05 | 3.21E+03 | 2.51E+04 | 1.13E+03 |
| pmb0713 | Tricin 7-O-hexosyl-O-hexoside | 1.79E+05 | 8.85E+03 | 1.25E+04 | 7.32E+03 |
| pmb0720 | Tricin O-malonylhexoside | 2.64E+05 | 1.45E+04 | 6.79E+04 | 1.28E+04 |
| pmb0724 | Tricin O-rhamnoside | 2.10E+05 | 1.39E+04 | 9.00E+00 | 0.00E+00 |
| pmb0736 | Tricin 7-O-hexoside | 6.30E+06 | 2.62E+05 | 5.16E+05 | 4.91E+04 |
| pmb2954 | Luteolin O-hexosyl-O-hexosyl-O-hexoside | 7.46E+05 | 6.81E+04 | 2.64E+06 | 3.29E+05 |
| pmb2986 | Chrysoeriol O-hexosyl-O-malonylhexoside | 1.97E+04 | 3.91E+03 | 8.45E+04 | 1.45E+04 |
| pmb2987 | Acacetin O-acetyl hexoside | 8.56E+05 | 6.67E+04 | 3.90E+07 | 4.43E+06 |
| pmb3006 | Apigenin 7-O-glucoside | 7.44E+04 | 7.02E+03 | 7.42E+04 | 2.24E+03 |
| pmb3041 | Tricin O-saccharic acid | 6.88E+05 | 5.95E+04 | 2.42E+06 | 8.29E+04 |
| pmb3042 | Tricin 5-O-hexoside | 2.79E+05 | 6.90E+03 | 3.67E+04 | 2.61E+03 |
| pmb3044 | Tricin di-O-hexoside | 3.39E+04 | 1.68E+03 | 2.74E+03 | 2.23E+03 |
| pme0089 | Luteolin | 3.37E+04 | 4.52E+03 | 9.00E+00 | 0.00E+00 |
| pme0324 | Chrysin | 5.25E+04 | 9.09E+03 | 9.00E+00 | 0.00E+00 |
| pme0359 | Apigenin 5-O-glucoside | 6.55E+05 | 4.52E+04 | 5.59E+05 | 2.57E+04 |
| pme0364 | Chrysoeriol | 1.27E+05 | 2.71E+04 | 4.69E+05 | 7.16E+04 |
| pme1518 | Nobiletin | 8.87E+04 | 6.94E+03 | 1.06E+05 | 7.36E+03 |
| pme1550 | Tangeretin | 6.98E+04 | 3.12E+03 | 7.93E+04 | 4.41E+03 |
| pme1611 | Isohemiphloin | 3.17E+04 | 3.05E+03 | 4.14E+04 | 5.83E+03 |
| pme1662 | sakuranetin | 9.00E+00 | 0.00E+00 | 1.01E+05 | 7.41E+03 |
| pme2459 | Luteolin 7-O-glucoside | 7.44E+06 | 9.46E+05 | 1.85E+07 | 4.37E+06 |
| pme3303 | Tricetin | 3.14E+05 | 6.43E+04 | 3.32E+04 | 4.75E+03 |
| pme3475 | Butin | 6.70E+06 | 4.43E+05 | 2.88E+06 | 1.69E+05 |
| pma0214 | methylQuercetin O-hexoside | 7.20E+06 | 9.80E+05 | 9.26E+06 | 1.30E+06 |
| pma6639 | Isorhamnetin O-hexoside | 3.92E+06 | 1.73E+06 | 1.14E+07 | 1.41E+06 |
| pmb0595 | Isorhamnetin 5-O-hexoside | 1.27E+07 | 1.40E+06 | 1.32E+07 | 2.12E+06 |
| pmb0711 | Quercetin 7-O-rutinoside | 9.65E+05 | 1.45E+05 | 1.89E+06 | 3.27E+05 |
| pmb3026 | Quercetin O-acetylhexoside | 2.56E+03 | 2.08E+03 | 4.61E+03 | 3.75E+03 |
| pmb3894 | Di-O-methylquercetin | 1.35E+06 | 5.74E+05 | 5.44E+05 | 1.39E+05 |
| pme0197 | Quercetin 3-O-rutinoside | 1.83E+06 | 1.86E+05 | 1.46E+06 | 2.46E+05 |
| pme0200 | Kaempferol | 8.91E+04 | 1.92E+04 | 5.28E+04 | 1.03E+04 |
| pme0361 | Quercetin 3-alpha-L-arabinofuranoside | 1.53E+05 | 2.84E+04 | 2.68E+05 | 3.41E+04 |
| pme0370 | Kaempferol 3-O-rutinoside | 2.05E+07 | 3.17E+06 | 4.60E+07 | 9.08E+06 |
| pme1480 | Myricetin | 3.68E+06 | 1.88E+05 | 9.00E+00 | 0.00E+00 |
| pme1502 | Kumatakenin | 9.00E+00 | 0.00E+00 | 5.42E+04 | 1.20E+04 |
| pme1521 | Dihydroquercetin | 1.99E+05 | 3.26E+04 | 3.78E+05 | 4.03E+04 |
| pme1539 | Isorhamnetin 3-O-neohesperidoside | 2.56E+06 | 2.06E+05 | 2.64E+06 | 3.95E+05 |
| pme1552 | Myricetin 3-O-rhamnoside | 3.25E+05 | 9.46E+03 | 9.00E+00 | 0.00E+00 |
| pme1590 | Isorhamnetin | 1.16E+04 | 6.24E+03 | 1.26E+04 | 1.18E+03 |
| pme1606 | Kaempferol 3-O-robinobioside | 1.99E+07 | 3.52E+06 | 4.69E+07 | 9.45E+06 |
| pme1622 | Kaempferol 3-O-glucoside | 5.99E+04 | 9.55E+03 | 1.72E+05 | 3.40E+04 |
| pme2898 | Dihydromyricetin | 2.20E+05 | 1.32E+04 | 1.11E+06 | 6.87E+04 |
| pme2954 | Quercetin | 1.23E+05 | 1.00E+05 | 3.97E+04 | 3.24E+04 |
| pme2963 | Aromadedrin | 4.52E+05 | 9.80E+04 | 3.73E+05 | 6.52E+04 |
| pme3130 | Quercetin 4'-O-glucoside | 1.79E+07 | 2.61E+06 | 6.92E+07 | 8.87E+06 |
| pme3211 | Quercetin 3-O-glucoside | 1.07E+06 | 2.20E+05 | 4.19E+06 | 4.91E+05 |
| pme3268 | Kaempferol 3-O-galactoside | 8.53E+06 | 1.07E+06 | 2.07E+07 | 4.55E+06 |
| pme3404 | Syringetin | 8.88E+04 | 1.83E+04 | 9.00E+00 | 0.00E+00 |
| pme3407 | Laricitrin | 1.31E+04 | 6.89E+03 | 9.00E+00 | 0.00E+00 |
| pme3442 | Quercetin 7-O-β-D-Glucuronide | 7.87E+03 | 6.42E+03 | 2.49E+04 | 3.33E+03 |
| pme3469 | Kaempferol-3-O-robinoside-7-O-rhamnoside | 3.12E+04 | 6.20E+03 | 4.01E+05 | 1.86E+04 |
| pme3484 | Myricetin 3-O-galactoside | 6.00E+04 | 2.24E+03 | 9.00E+00 | 0.00E+00 |
| pme3514 | Morin | 4.03E+05 | 1.88E+05 | 1.28E+05 | 3.48E+04 |
| pma0724 | Naringenin C-hexoside | 1.39E+05 | 1.63E+04 | 1.89E+05 | 2.42E+04 |
| pma6218 | O-methylnaringenin C-pentoside | 3.35E+05 | 1.67E+04 | 1.54E+06 | 7.37E+04 |
| pma6496 | Luteolin 6-C-glucoside | 8.57E+03 | 6.99E+03 | 1.32E+05 | 5.58E+03 |
| pma6516 | C-hexosyl-apigenin O-hexosyl-O-hexoside | 2.22E+05 | 1.96E+04 | 8.30E+04 | 4.71E+03 |
| pmb0378 | Luteolin O-feruloylhexoside | 1.94E+04 | 7.90E+03 | 8.91E+04 | 3.18E+04 |
| pmb0615 | Hesperetin C-hexosyl-O-hexosyl-O-hexoside | 3.12E+06 | 2.01E+05 | 2.70E+05 | 2.76E+04 |
| pmb0618 | 8-C-hexosyl-hesperetin O-hexoside | 4.59E+05 | 5.40E+04 | 1.05E+07 | 7.89E+05 |
| pmb0619 | Eriodictiol 6-C-hexoside 8-C-hexoside-O-hexoside | 1.87E+05 | 2.41E+04 | 2.87E+06 | 3.65E+05 |
| pmb0620 | Chrysoeriol 6-C-hexoside 8-C-hexoside-O-hexoside | 2.89E+05 | 2.02E+04 | 1.76E+04 | 2.03E+03 |
| pmb0623 | 6-C-hexosyl chrysoeriol O-hexoside | 6.92E+04 | 8.73E+03 | 4.58E+04 | 1.73E+03 |
| pmb0624 | 6-C-hexosyl-luteolin O-hexoside | 2.93E+04 | 4.25E+03 | 1.01E+05 | 5.55E+03 |
| pmb0626 | 6-C-hexosyl-apigenin O-hexosyl-O-hexoside | 9.00E+00 | 0.00E+00 | 3.92E+04 | 5.54E+03 |
| pmb0628 | Eriodictiol C-hexosyl-O-hexoside | 2.91E+04 | 5.04E+02 | 1.37E+06 | 1.58E+05 |
| pmb0635 | C-hexosyl-apigenin O-feruloylhexoside-O-hexoside | 5.44E+05 | 7.66E+03 | 9.00E+00 | 0.00E+00 |
| pmb0639 | 8-C-hexosyl-apigenin O-hexosyl-O-hexoside | 5.84E+04 | 2.47E+04 | 4.60E+05 | 4.39E+04 |
| pmb0645 | 6-C-hexosyl-hesperetin O-hexoside | 1.14E+06 | 1.03E+05 | 6.91E+05 | 8.47E+04 |
| pmb0660 | C-hexosyl-luteolin O-p-coumaroylhexoside | 1.89E+05 | 1.47E+04 | 9.00E+00 | 0.00E+00 |
| pmb0661 | Chrysoeriol C-hexosyl-O-rhamnoside | 8.44E+04 | 1.30E+04 | 1.69E+05 | 2.72E+04 |
| pmb0662 | C-hexosyl-luteolin O-feruloylhexoside | 1.03E+05 | 8.11E+03 | 9.00E+00 | 0.00E+00 |
| pmb0665 | Luteolin 8-C-hexosyl-O-hexoside | 2.12E+06 | 3.93E+05 | 4.18E+06 | 7.31E+05 |
| pmb0666 | 6-C-hexosyl-apigenin O-sinapoylhexoside | 4.73E+05 | 8.73E+03 | 9.00E+00 | 0.00E+00 |
| pmb0672 | 6-C-hexosyl-apigenin O-feruloylhexoside | 9.84E+05 | 4.57E+04 | 9.00E+00 | 0.00E+00 |
| pmb0680 | C-hexosyl-apigenin O-p-coumaroylhexoside | 1.94E+05 | 1.98E+04 | 9.00E+00 | 0.00E+00 |
| pmb0691 | Luteolin C-hexosyl-O-rhamnoside O-hexoside | 3.37E+05 | 3.22E+04 | 1.61E+05 | 4.32E+03 |
| pmb2969 | Hesperetin C-hexoside O-hexoside | 1.50E+04 | 9.53E+02 | 9.00E+00 | 0.00E+00 |
| pmb3023 | Eriodictyol C-hexoside | 6.52E+04 | 1.01E+04 | 1.24E+06 | 2.67E+05 |
| pme1624 | Isovitexin | 6.84E+03 | 5.58E+03 | 3.26E+04 | 3.81E+03 |
| pme3227 | Vitexin 2''-O-beta-L-rhamnoside | 9.00E+00 | 0.00E+00 | 3.34E+03 | 2.72E+03 |
| pma0791 | Naringenin O-malonylhexoside | 1.33E+05 | 2.10E+04 | 1.49E+05 | 1.50E+04 |
| pmb2970 | Hesperetin O-hexosyl-O-hexoside | 2.30E+05 | 1.66E+04 | 9.00E+00 | 0.00E+00 |
| pmb2979 | Hesperetin O-malonylhexoside | 3.86E+04 | 8.81E+02 | 1.49E+05 | 4.97E+03 |
| pmc1990 | "4'-Hydroxy-5,7-dimethoxyflavanone" | 9.26E+04 | 2.52E+04 | 7.46E+04 | 6.68E+03 |
| pme0002 | Hesperetin 7-O-neohesperidoside | 4.88E+06 | 1.39E+06 | 5.99E+06 | 1.36E+06 |
| pme0331 | Naringenin 7-O-neohesperidoside | 1.54E+06 | 1.21E+05 | 2.14E+05 | 2.44E+04 |
| pme0372 | Naringenin 7-O-glucoside | 3.24E+06 | 8.33E+04 | 1.51E+06 | 1.35E+05 |
| pme0377 | Naringenin | 6.83E+06 | 4.62E+05 | 2.94E+06 | 1.74E+05 |
| pme0421 | Phloretin | 1.62E+05 | 1.81E+04 | 6.11E+04 | 3.75E+03 |
| pme1399 | Xanthohumol | 1.82E+04 | 8.77E+02 | 2.45E+04 | 3.74E+02 |
| pme1583 | Eriodictyol | 2.19E+05 | 7.07E+04 | 7.44E+06 | 2.34E+05 |
| pme1598 | Hesperetin 5-O-glucoside | 1.32E+06 | 1.68E+05 | 4.84E+06 | 5.76E+05 |
| pme2321 | Hesperetin | 5.65E+05 | 6.55E+04 | 5.89E+06 | 4.23E+05 |
| pme2950 | Hesperetin 7-rutinoside | 4.81E+06 | 1.60E+06 | 4.74E+06 | 1.57E+06 |
| pme2960 | Naringenin chalcone | 6.75E+06 | 3.99E+05 | 2.95E+06 | 1.72E+05 |
| pme2982 | Dihydrochrysin | 3.85E+05 | 2.00E+05 | 9.00E+00 | 0.00E+00 |
| pme3282 | "Afzelechin | 4.31E+04 | 6.74E+02 | 5.16E+04 | 2.14E+03 |
| pme3461 | Homoeriodictyol | 5.24E+04 | 6.62E+03 | 5.90E+05 | 5.07E+04 |
| pme3466 | Isosakuranetin | 9.00E+00 | 0.00E+00 | 6.88E+04 | 1.44E+04 |
| pme1587 | Daidzein 7-O-glucoside | 9.00E+00 | 0.00E+00 | 4.10E+05 | 3.29E+04 |
| pme3208 | Glycitin | 9.00E+00 | 0.00E+00 | 1.74E+04 | 2.17E+03 |
| pme3210 | Genistein 7-O-Glucoside | 5.81E+05 | 4.51E+04 | 5.66E+05 | 4.90E+04 |
| pme3279 | 2'-Hydroxygenistein | 5.32E+04 | 6.04E+03 | 9.00E+00 | 0.00E+00 |
| pme3399 | Sissotrin | 4.79E+03 | 2.04E+03 | 9.00E+00 | 0.00E+00 |
| pme3502 | Formononetin 7-O-glucoside | 2.50E+05 | 4.27E+04 | 2.07E+04 | 6.98E+02 |

**Supplementary Table S5**

Summary of *L. ruthenicum* clean reads

| Sample | Raw Reads | Clean Reads | Clean Bases | Error (%) | Q20(%) | Q30(%) | GC Content (%) |
| --- | --- | --- | --- | --- | --- | --- | --- |
| B1_I | 60639374 | 57502430 | 8.63G | 0.01 | 97.8 | 94.37 | 43.55 |
| B1_II | 65944932 | 62534666 | 9.38G | 0.01 | 97.75 | 94.26 | 43.31 |
| B1_III | 60177852 | 56802102 | 8.52G | 0.01 | 97.92 | 94.63 | 43.15 |
| B2_I | 71826238 | 68171678 | 10.23G | 0.01 | 97.87 | 94.52 | 43.07 |
| B2_II | 69295054 | 65842044 | 9.88G | 0.01 | 97.86 | 94.51 | 43.08 |
| B2_III | 63122364 | 59945726 | 8.99G | 0.01 | 97.82 | 94.43 | 43.2 |
| B3_I | 65592602 | 62247288 | 9.34G | 0.01 | 97.86 | 94.51 | 42.72 |
| B3_II | 62982618 | 62073336 | 9.31G | 0.01 | 98.05 | 94.95 | 42.49 |
| B3_III | 64386996 | 63449252 | 9.52G | 0.01 | 97.98 | 94.8 | 42.66 |
| B4_I | 61857704 | 60951390 | 9.14G | 0.01 | 97.99 | 94.8 | 42.11 |
| B4_II | 69043300 | 67981522 | 10.2G | 0.01 | 97.89 | 94.58 | 42.05 |
| B4_III | 74384656 | 73227696 | 10.98G | 0.01 | 97.79 | 94.39 | 42.04 |
| B5_I | 63452634 | 60945380 | 9.14G | 0.01 | 97.55 | 93.79 | 42.22 |
| B5_II | 73736472 | 72709840 | 10.91G | 0.01 | 98.08 | 95.03 | 42.14 |
| B5_III | 51012960 | 50163092 | 7.52G | 0.01 | 98.21 | 95.3 | 42.29 |
| w1_I | 52499934 | 52465654 | 7.87G | 0.01 | 98.43 | 95.78 | 42.67 |
| w1_II | 52816702 | 52782116 | 7.92G | 0.01 | 98.4 | 95.71 | 42.27 |
| w1_III | 53688868 | 53675438 | 8.05G | 0.01 | 97.18 | 92.86 | 42.09 |
| w2_I | 50687502 | 50674004 | 7.6G | 0.01 | 97.45 | 93.51 | 43.25 |
| w2_II | 49410210 | 49396926 | 7.41G | 0.01 | 97.47 | 93.56 | 43.47 |
| w2_III | 49057052 | 49043530 | 7.36G | 0.01 | 97.52 | 93.64 | 43.45 |
| w3_I | 47369802 | 47356860 | 7.1G | 0.01 | 97.52 | 93.61 | 43.17 |
| w3_II | 56249250 | 56233742 | 8.44G | 0.01 | 97.64 | 93.85 | 43.04 |
| w3_III | 47407068 | 47393954 | 7.11G | 0.01 | 97.59 | 93.77 | 43.1 |
| w4_I | 58429536 | 58414720 | 8.76G | 0.01 | 97.71 | 94.02 | 42.79 |
| w4_II | 54316416 | 54302210 | 8.15G | 0.01 | 97.66 | 93.88 | 43.76 |
| w4_III | 53956670 | 53942788 | 8.09G | 0.01 | 97.53 | 93.62 | 43.47 |
| w5_I | 54635830 | 54621142 | 8.19G | 0.01 | 97.51 | 93.59 | 43.48 |
| w5_II | 49466398 | 49452734 | 7.42G | 0.01 | 97.43 | 93.43 | 43.19 |
| w5_III | 52085416 | 52071244 | 7.81G | 0.01 | 97.29 | 93.13 | 43.13 |

**Supplementary Table S6**

Summary for annotation results of *L. ruthenicum* unigenes

|  | Number of Unigenes | Percentage (%) | |
| --- | --- | --- | --- |
| Annotated in NR | 150717 | 54.87 | |
| Annotated in NT | 161134 | 58.67 | |
| Annotated in KO | 57053 | 20.77 | |
| Annotated in SwissProt | 103372 | 37.63 | |
| Annotated in PFAM | 101154 | 36.83 | |
| Annotated in GO | 101876 | 37.09 | |
| Annotated in KOG | 37704 | 13.72 | |
| Annotated in all Databases | 23309 | 8.48 | |
| Annotated in at least one Database | 188308 | 68.56 | |
| Total Unigenes | 274634 | | 100 |
